# Supplementary material for: Long lifetimes white afterglow in slightly crosslinked polymer systems
Source: Nat Commun. 2024 Apr 5;15:2947. doi: 10.1038/s41467-024-47378-2 (PMC10997626; doi:10.1038/s41467-024-47378-2)
Supplement: Supplementary file 1 — Supplementary Information [file 41467_2024_47378_MOESM1_ESM.pdf]

Supplementary Information

Long lifetimes white afterglow in slightly crosslinked polymer systems

Chen et al.

Contents

Supplementary Figures 2

Supplementary Tables 31

Supplementary Figures

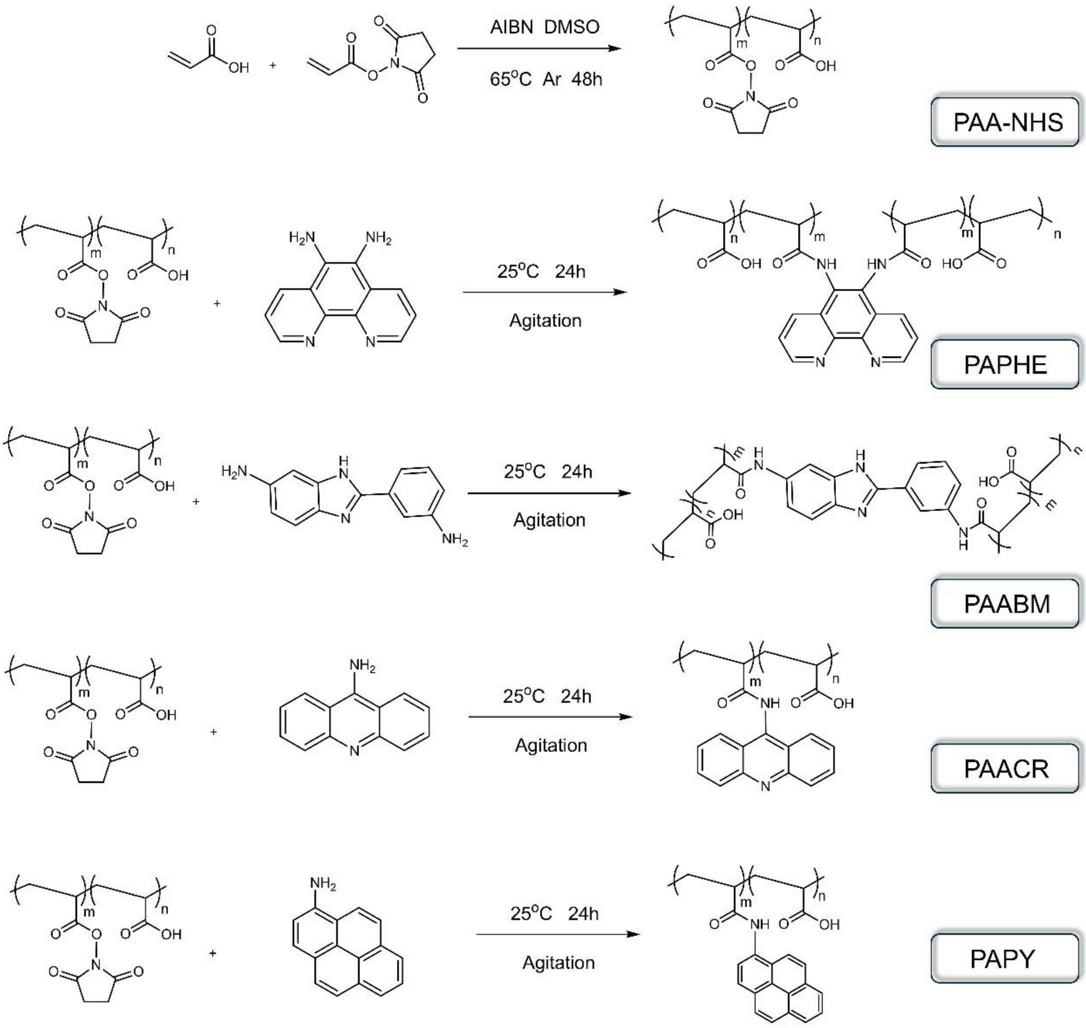

Supplementary Figure 1. Synthesis route of one-component polymer.

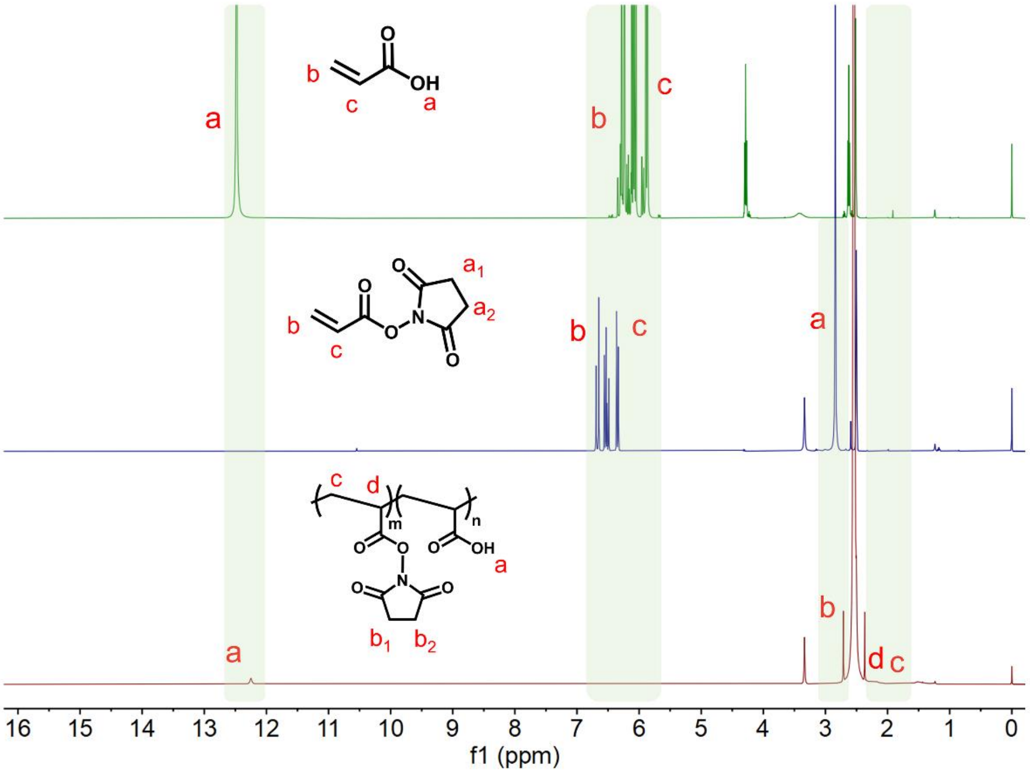

Supplementary Figure 2. ^{1}H NMR spectra of pure PAA-NHS in DMSO-d_6.

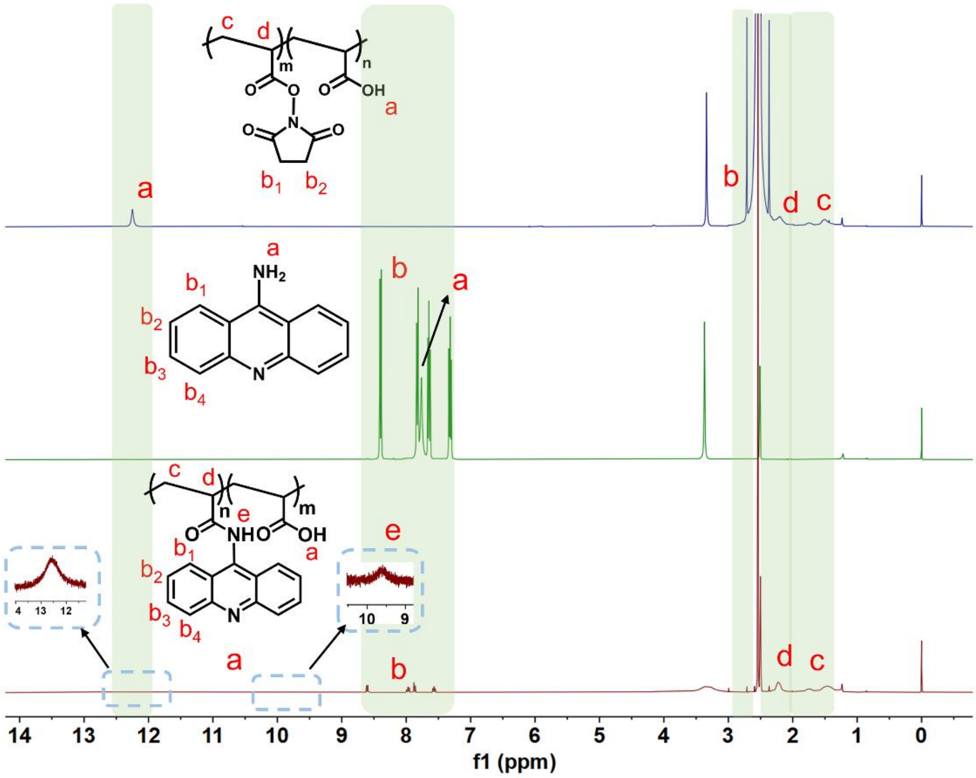

Supplementary Figure 3. ^{1}H NMR spectra of pure PAACR in DMSO-d_6.

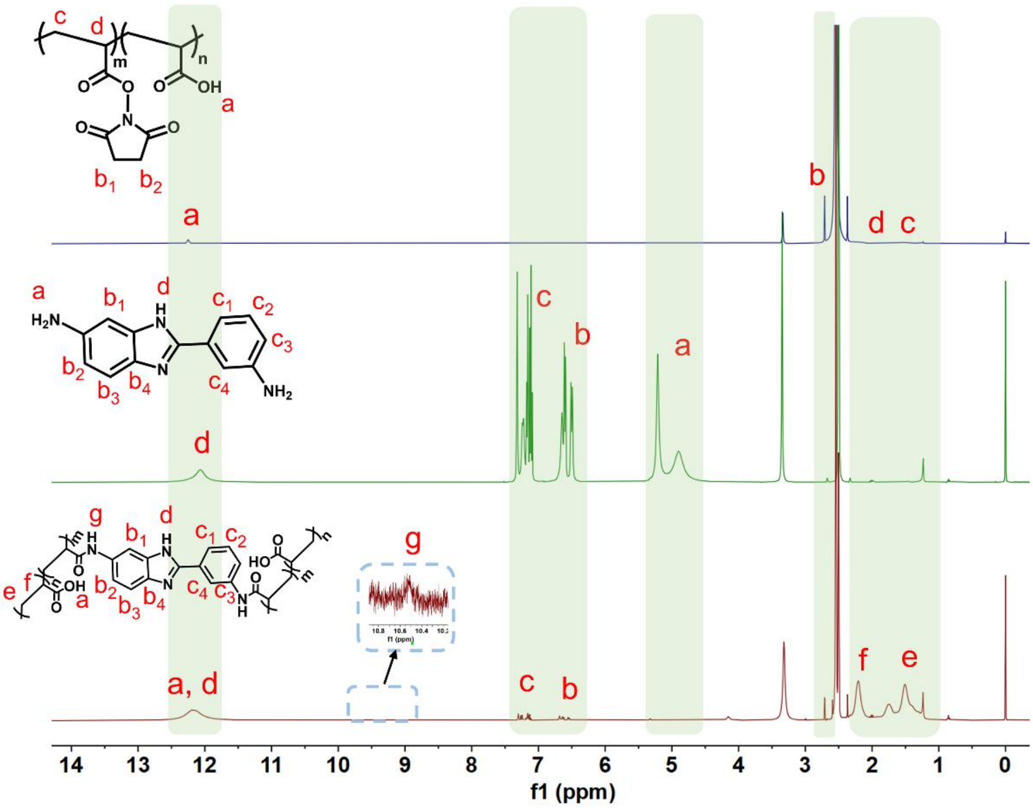

Supplementary Figure 4. ^{1}H NMR spectra of pure PAABM in DMSO-d_6.

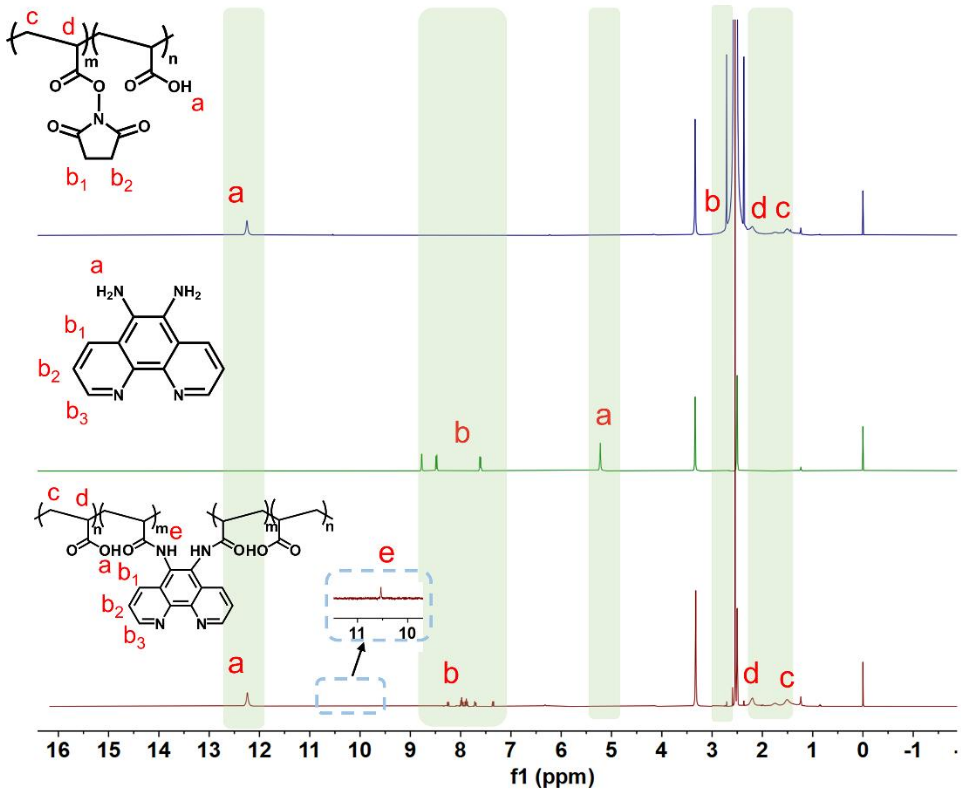

Supplementary Figure 5. ^{1}H NMR spectra of pure PAPHE in DMSO-d_6.

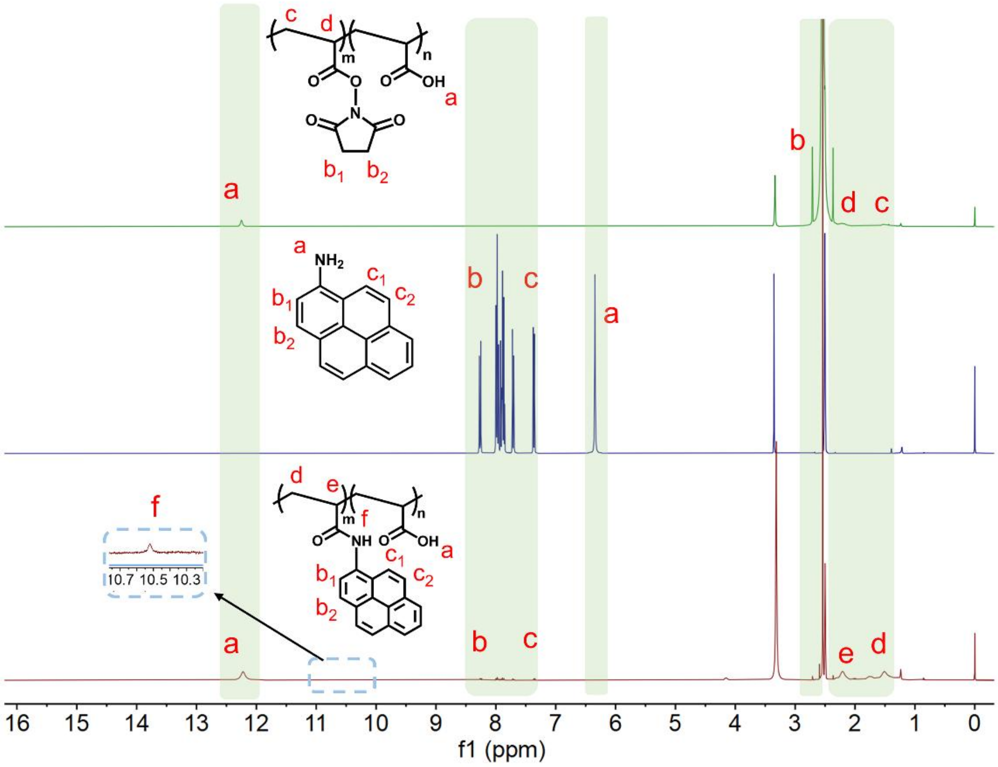

Supplementary Figure 6. ^{1}H NMR spectra of pure PAPY in DMSO-d_6.

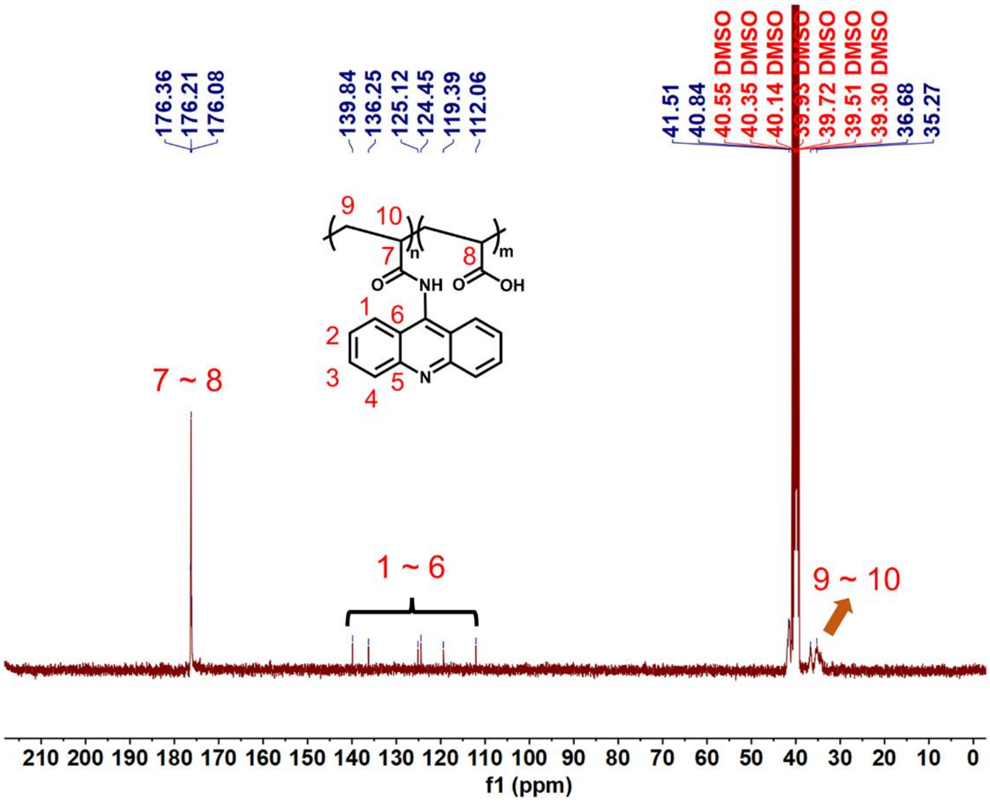

Supplementary Figure 7. ^{13}C NMR spectra of pure PAACR in DMSO-d_6.

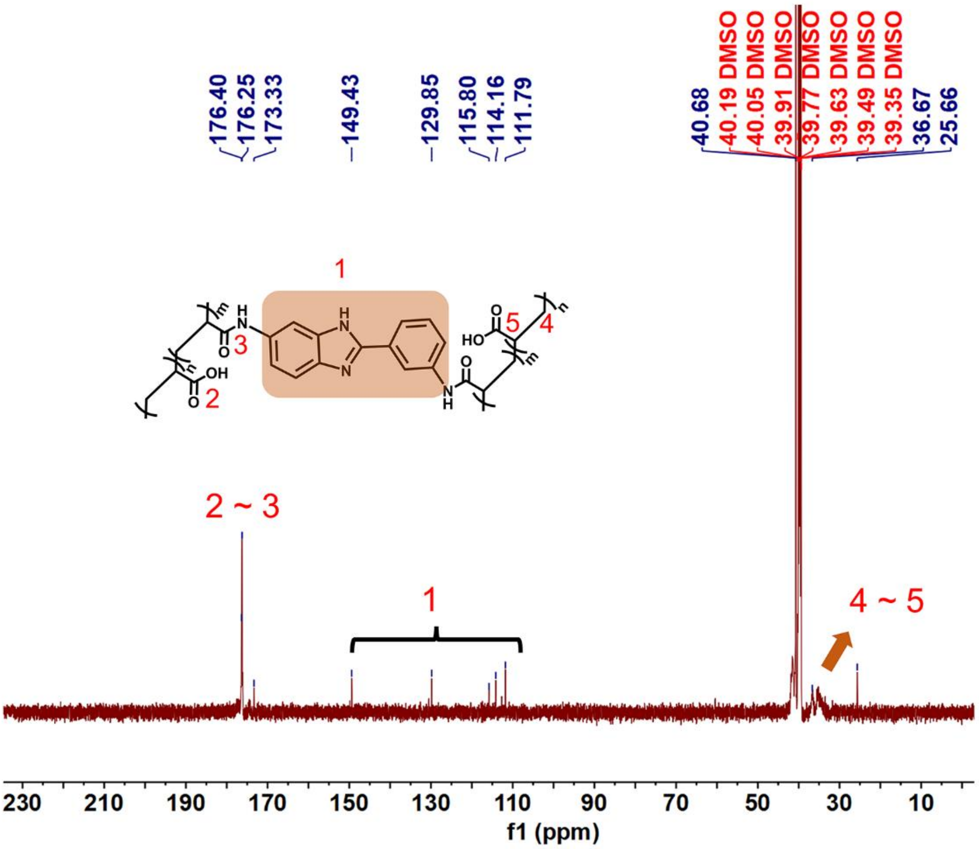

Supplementary Figure 8. ^{13}C NMR spectra of pure PAABM in DMSO-d_6.

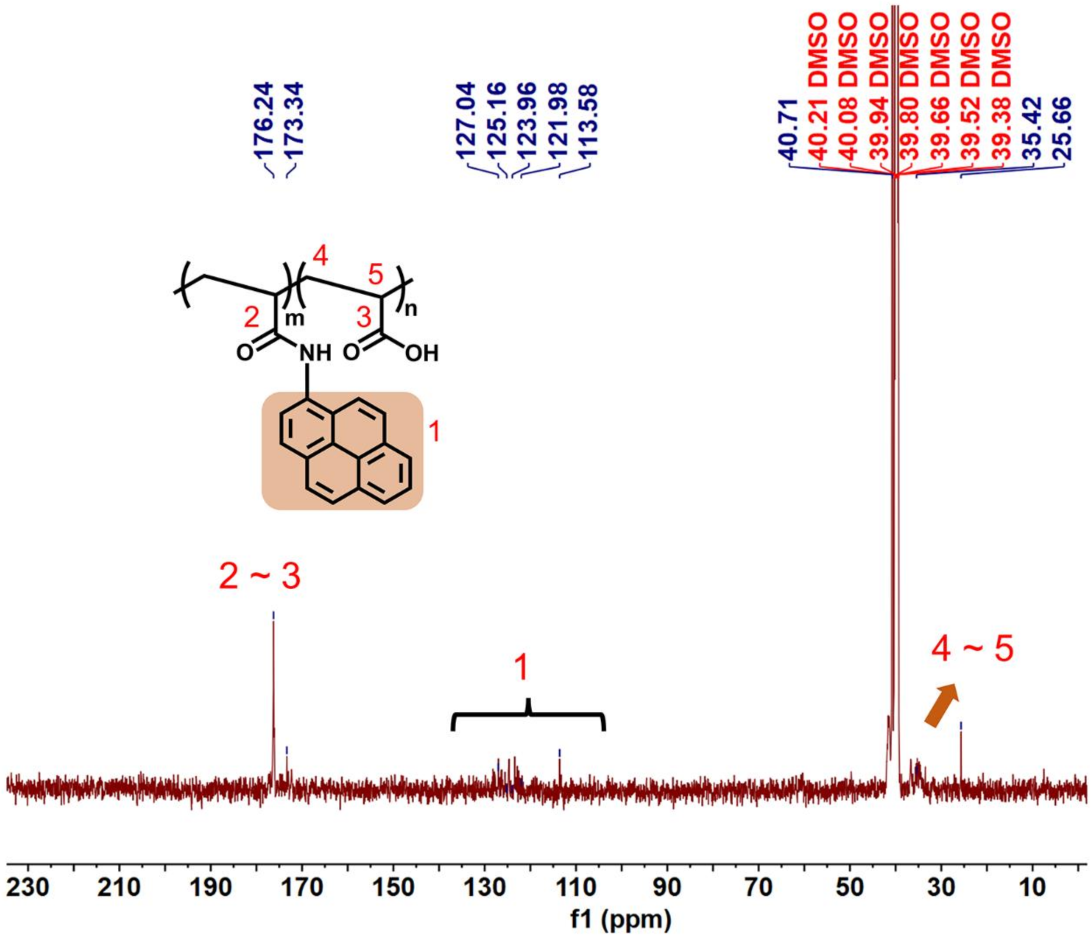

Supplementary Figure 9. ^{13}C NMR spectra of pure PAPY in DMSO-d_6.

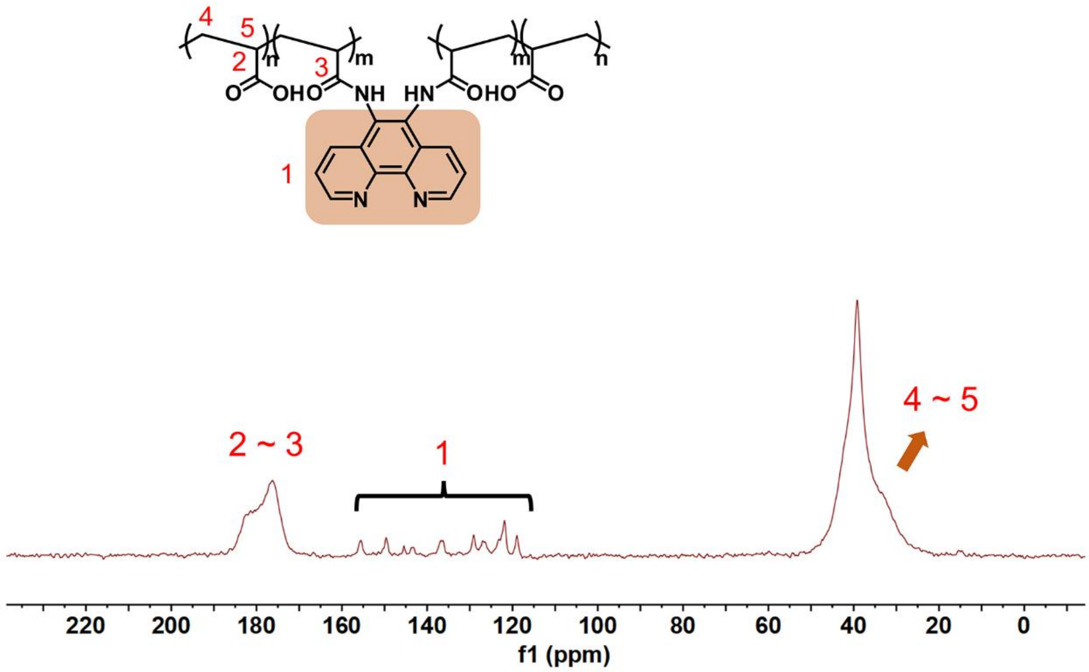

Supplementary Figure 10. Solid-state ^{13}C NMR spectra of PAPHE.

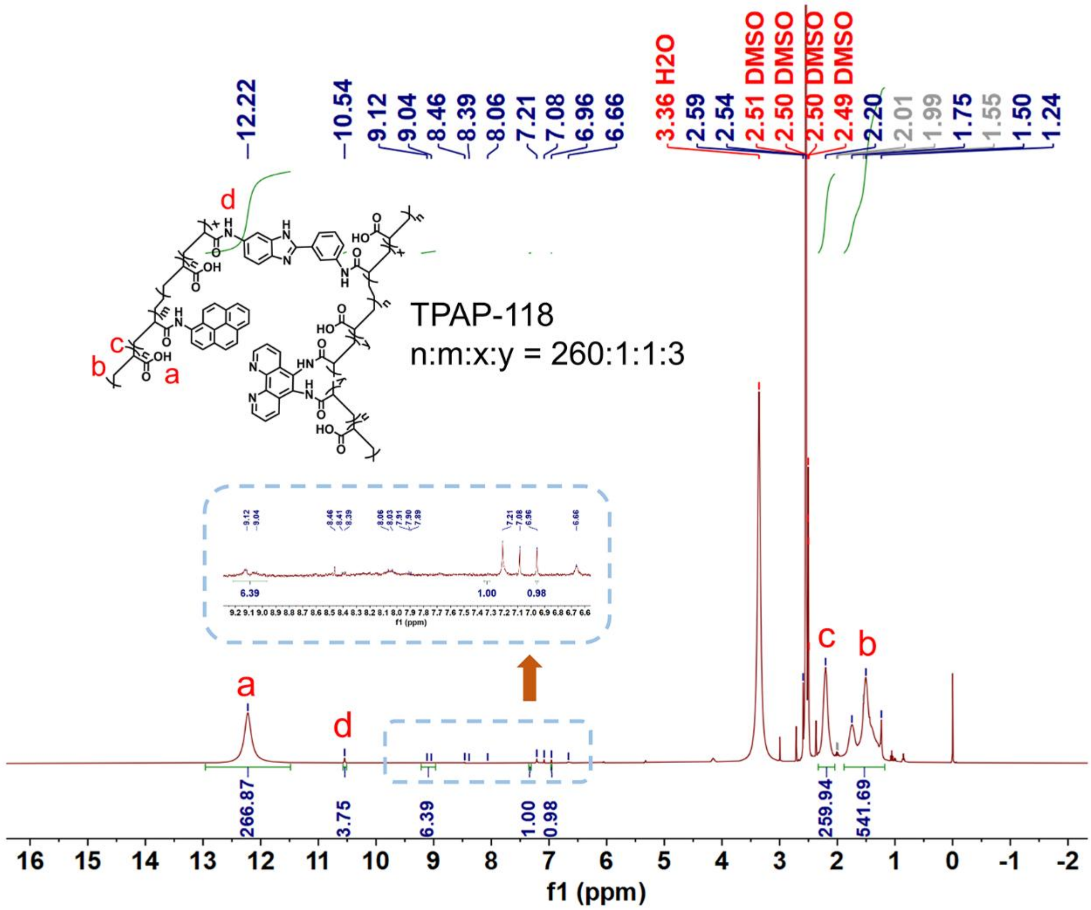

Supplementary Figure 11. ^{1}H NMR spectra of TPAP-118 in DMSO-d_6.

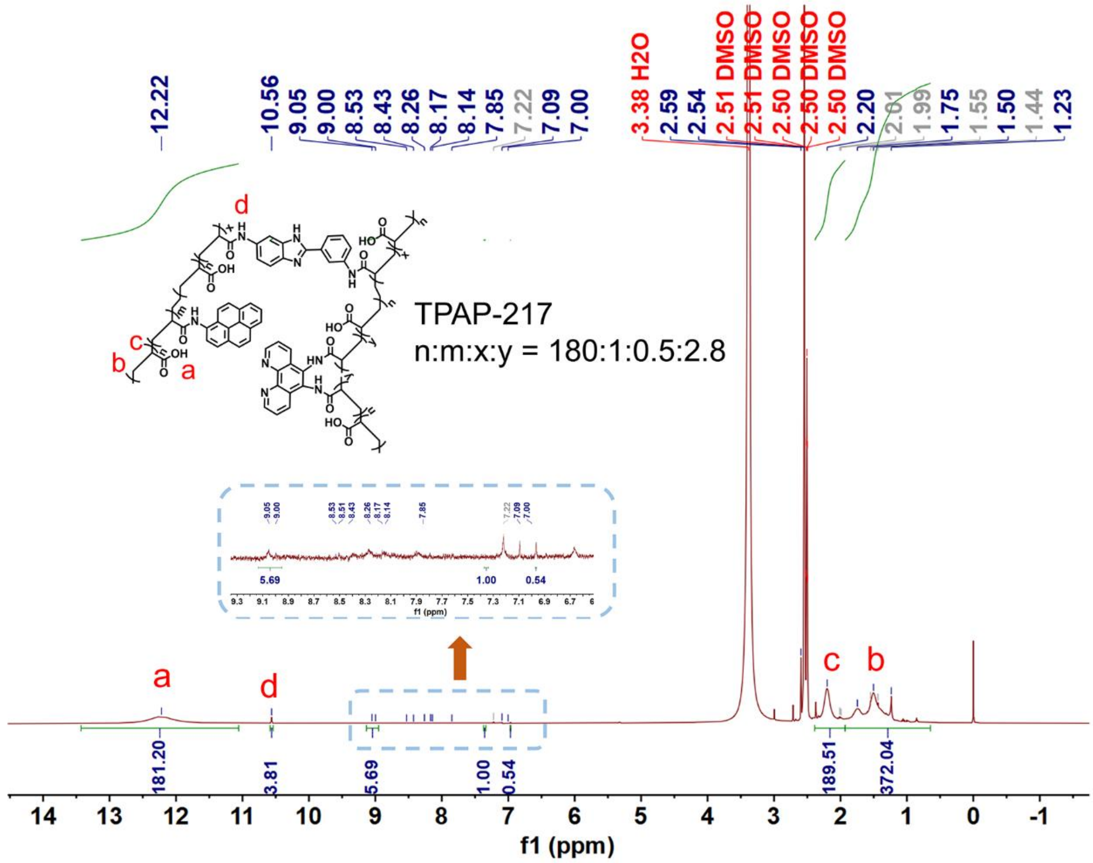

Supplementary Figure 12. ^{1}H NMR spectra of TPAP-217 in DMSO-d_6.

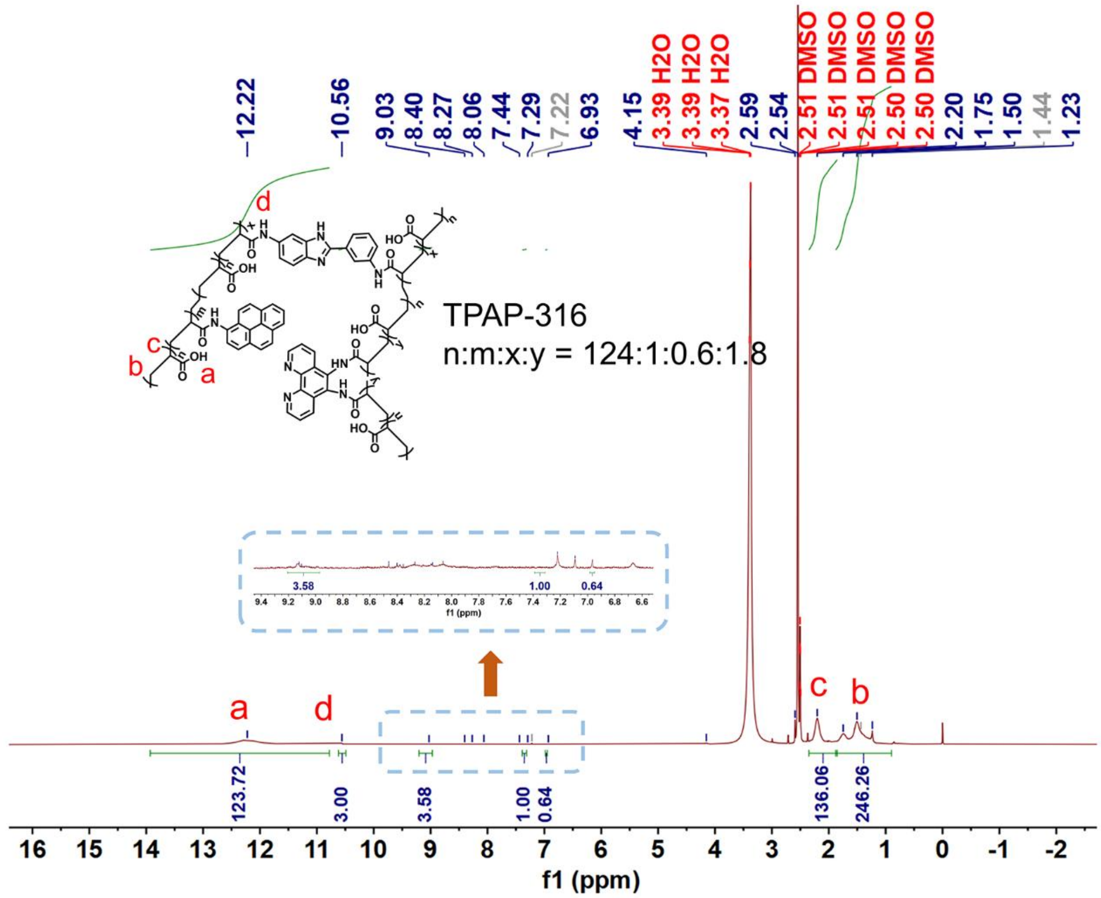

Supplementary Figure 13. ^{1}H NMR spectra of TPAP-316 in DMSO-d_6.

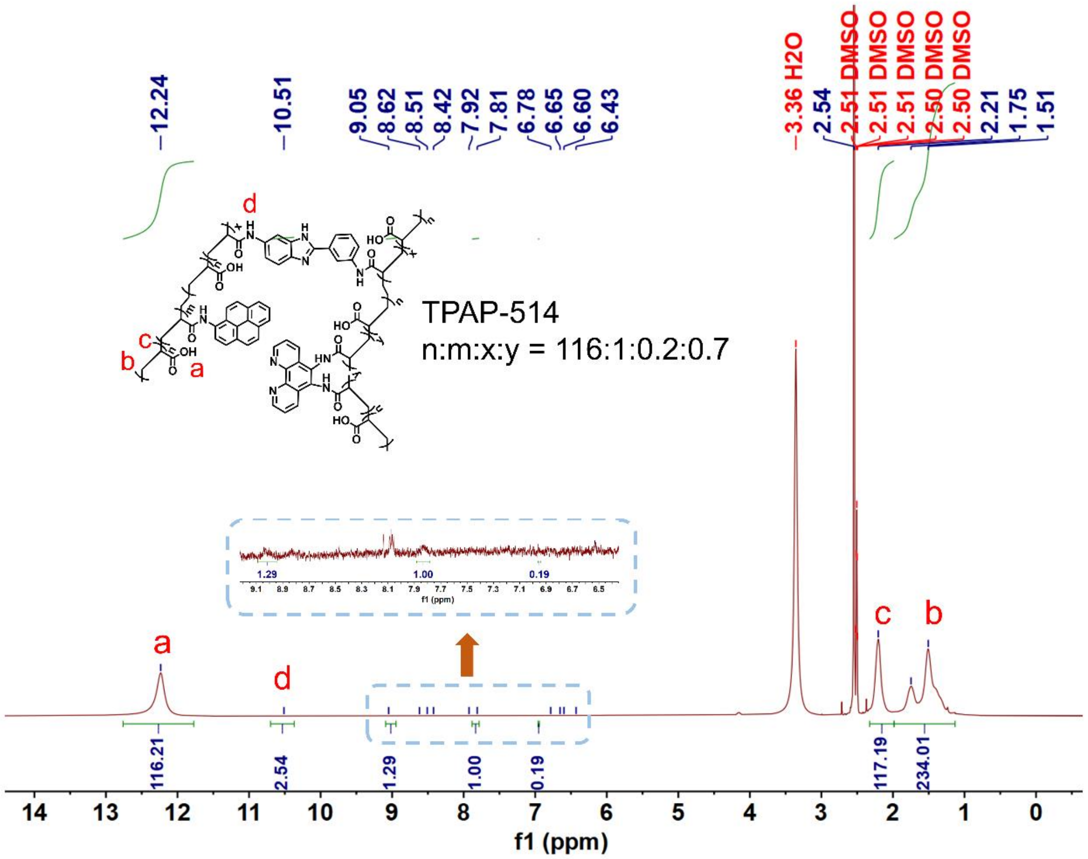

Supplementary Figure 14. ^{1}H NMR spectra of TPAP-514 in DMSO-d_6.

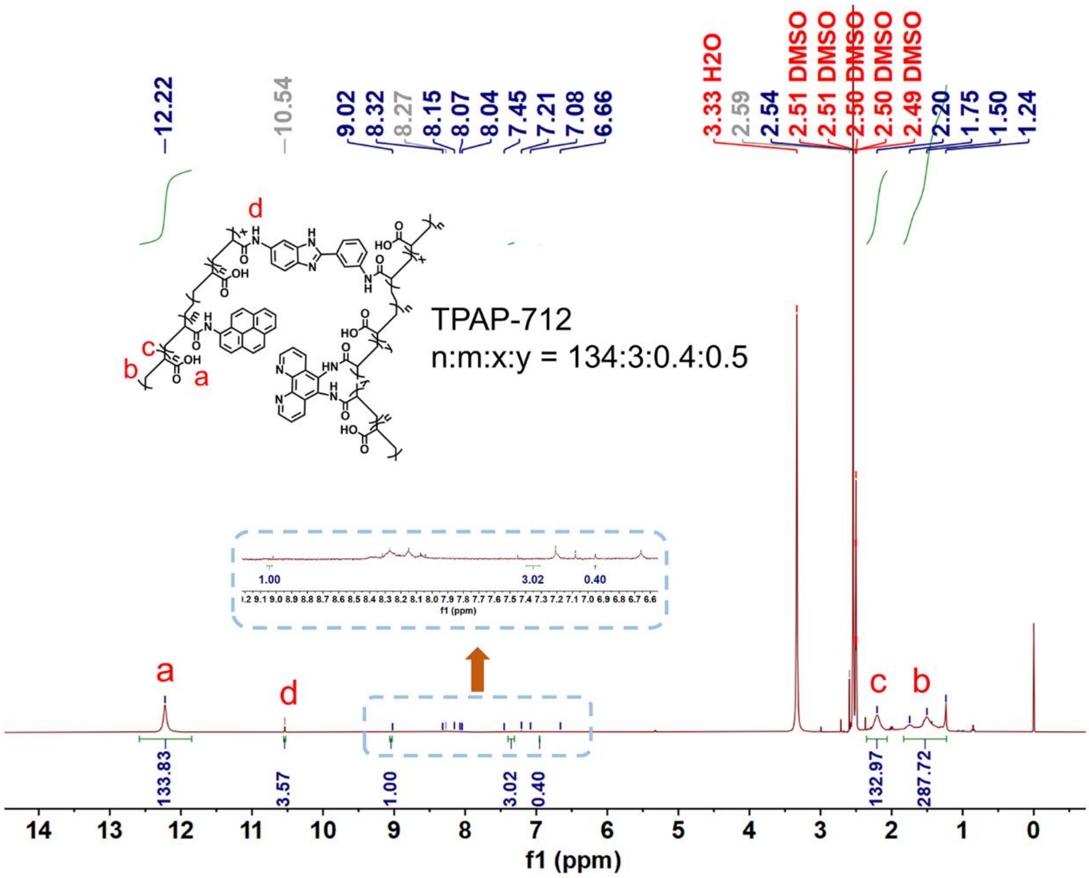

Supplementary Figure 15. ^{1}H NMR spectra of TPAP-712 in DMSO-d_6.

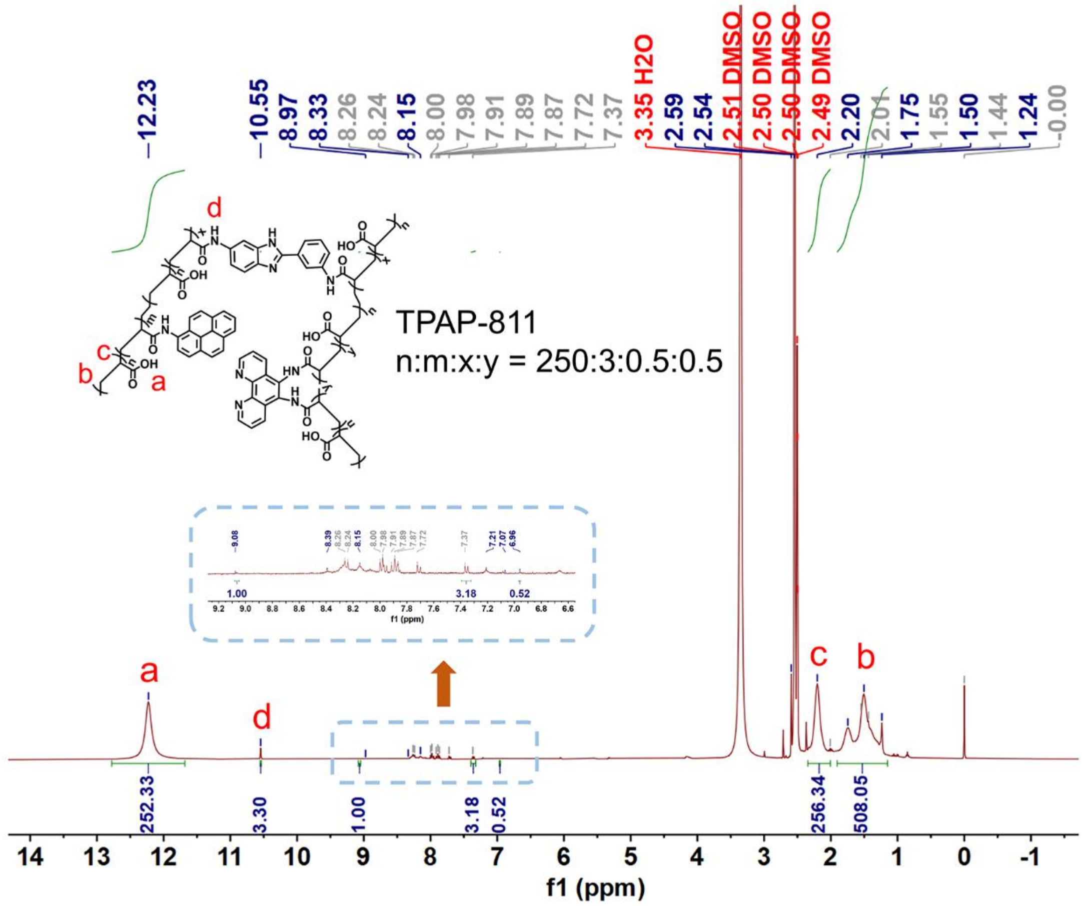

Supplementary Figure 16. ^{1}H NMR spectra of TPAP-811 in DMSO-d_6.

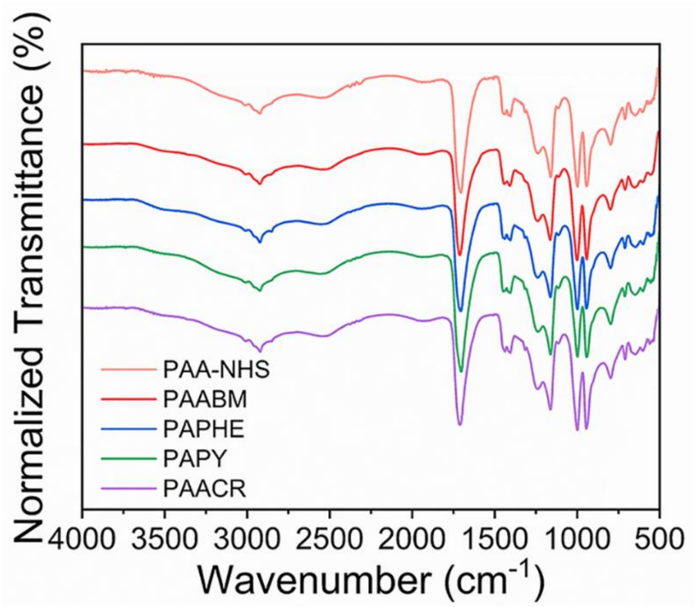

Supplementary Figure 17. FTIR spectra of films PAA-NHS, PAABM, PAPHE, PAPY and PAACR.

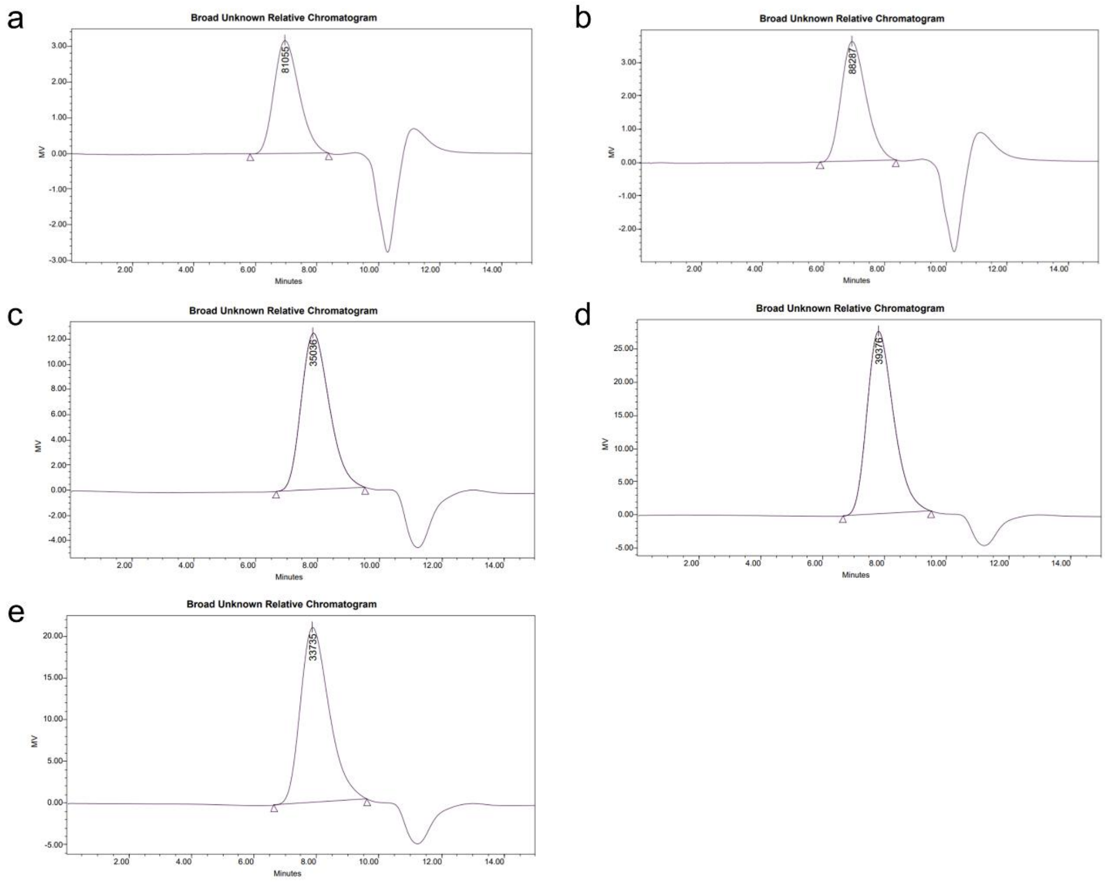

Supplementary Figure 18. a-e GPC curves of polymers TPAP-514 (**a**), PAPHE (**b**), PAABM (**c**), PAACR (**d**) and PAPY (**e**) using DMSO as the mobile phase.

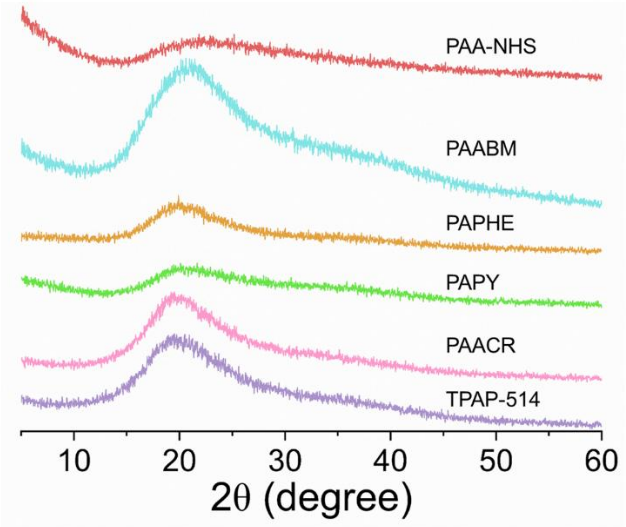

Supplementary Figure 19. X-ray diffraction patterns of PAA-NHS, one-component and three-component polymer films.

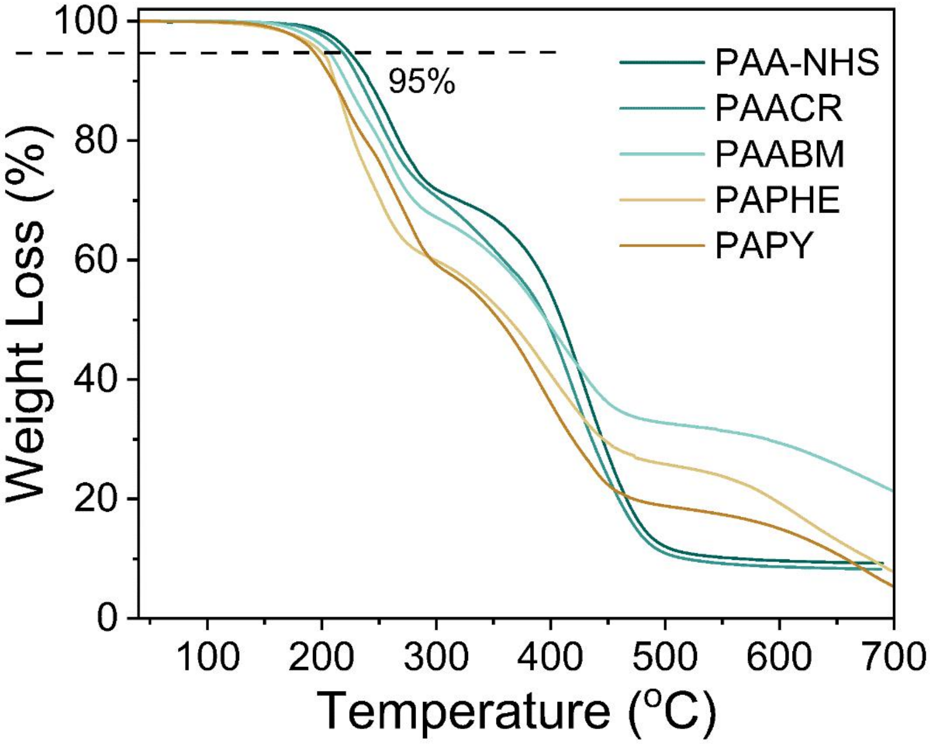

Supplementary Figure 20. Thermogravimetric analysis curves of PAA-NHS and all single-component polymers with heating rate of 10 °C/min under the N_2 atmosphere.

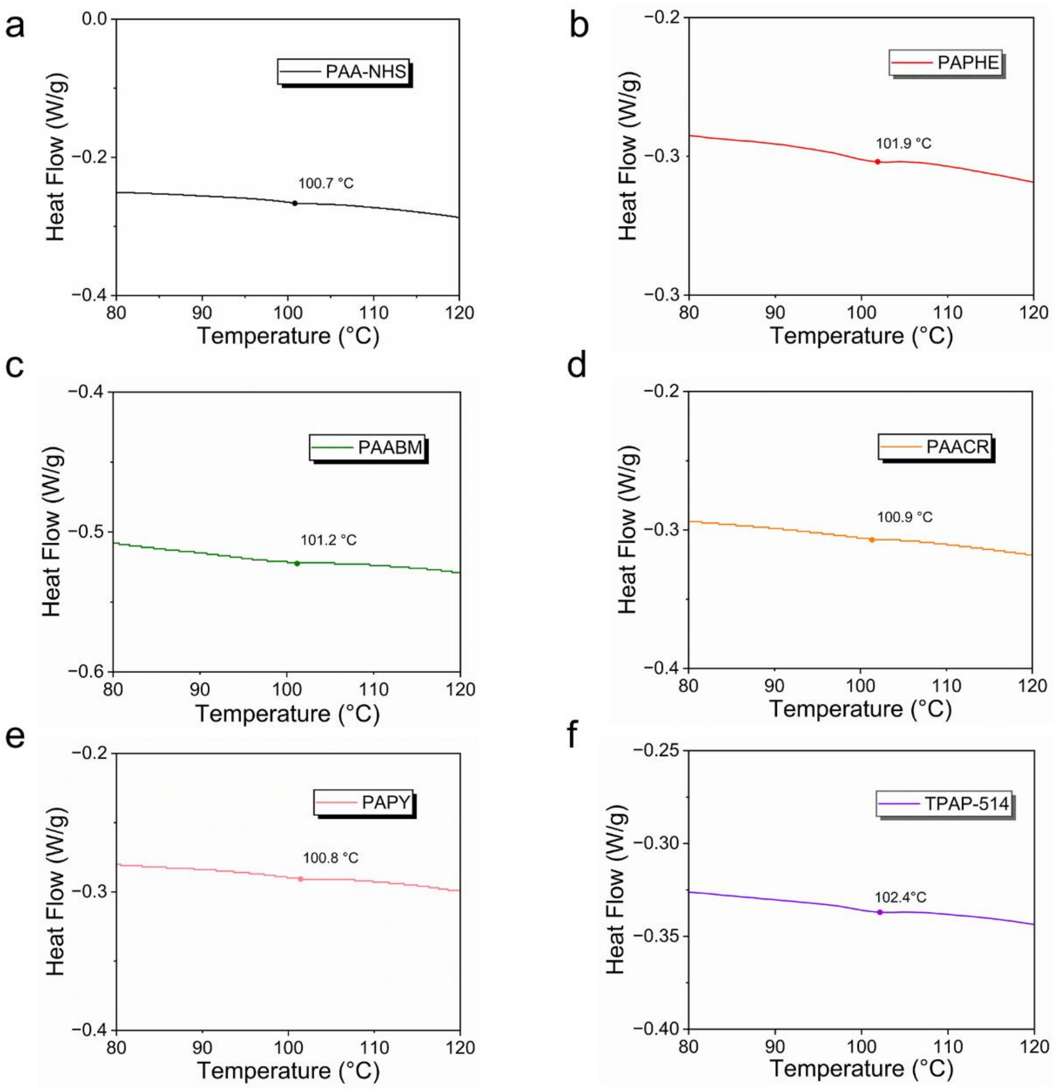

Supplementary Figure 21. a-f DSC curves of PAA-NHS (**a**), PAPHE (**b**), PAABM (**c**), PAACR (**d**), PAPY (**e**) and TPAP-514 (**f**).

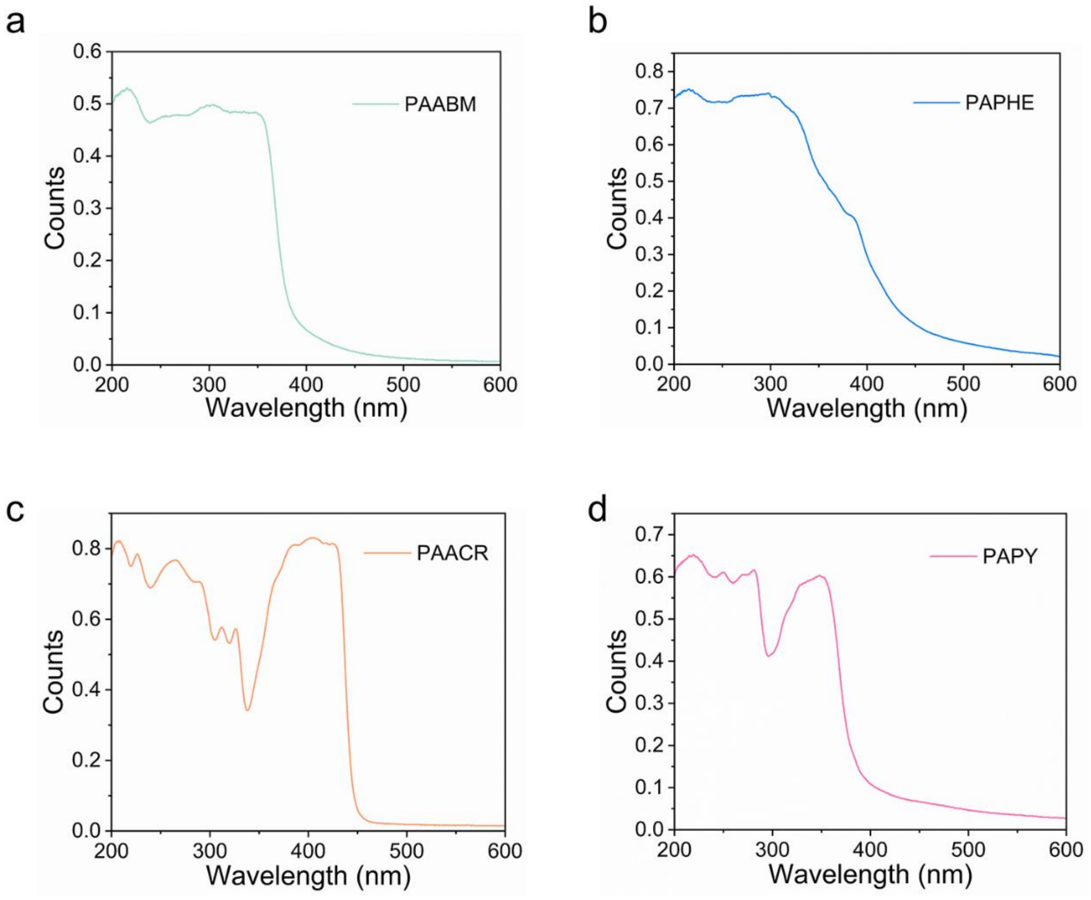

Supplementary Figure 22. a-d UV-Vis absorption spectra of films of PAABM (**a**), PAPHE (**b**), PAACR (**c**) and PAPY (**d**).

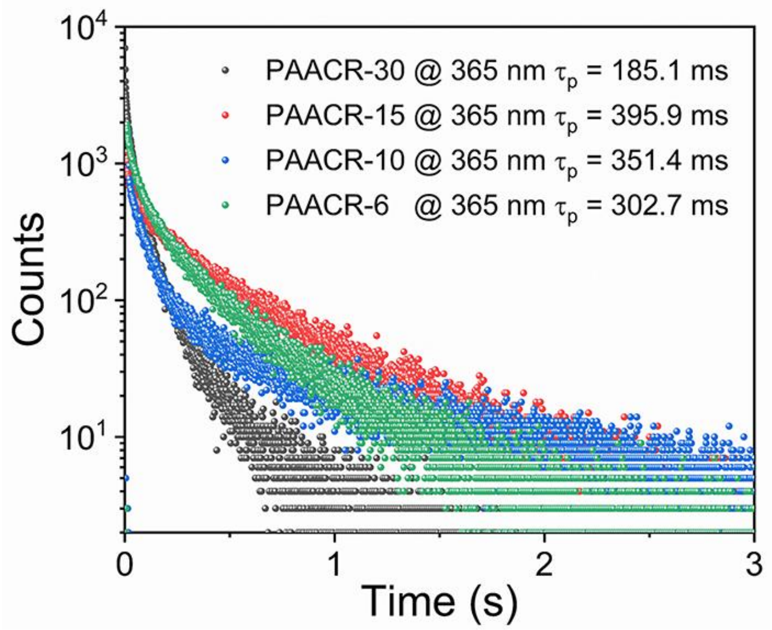

Supplementary Figure 23. Phosphorescence decay curves of PAACR films with different chromophore concentrations at 566 nm.

nm).

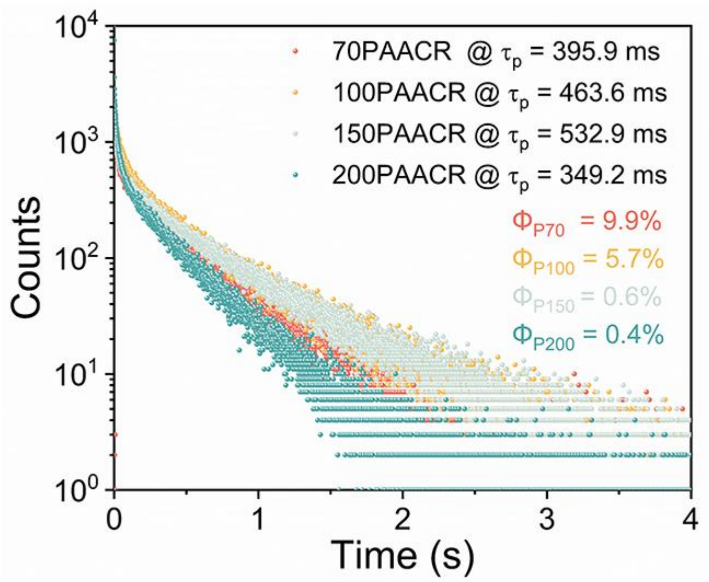

Supplementary Figure 24. The phosphorescence decay curve and phosphorescence quantum yield of PAACR films with different acrylic monomer content at 566 nm.

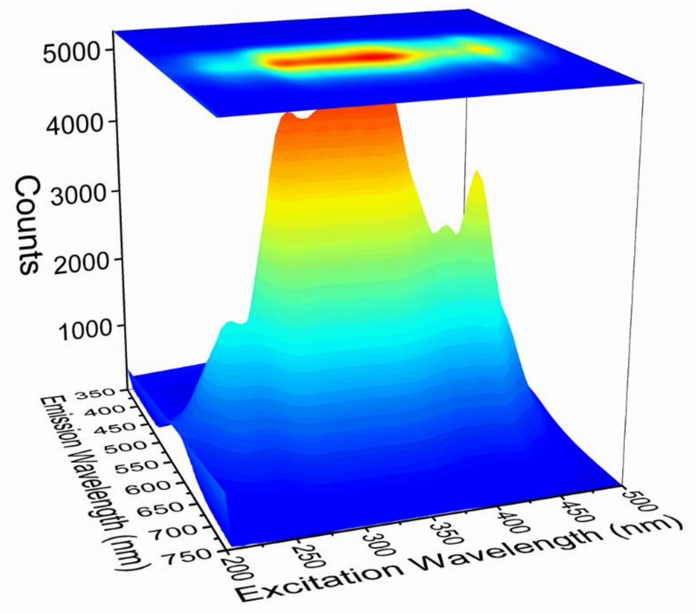

Supplementary Figure 25. Excitation-emission phosphorescence mapping of polymer films PAACR (excitation wavelength from 200 to 500 nm, emission wavelength from 350 to 750 nm).

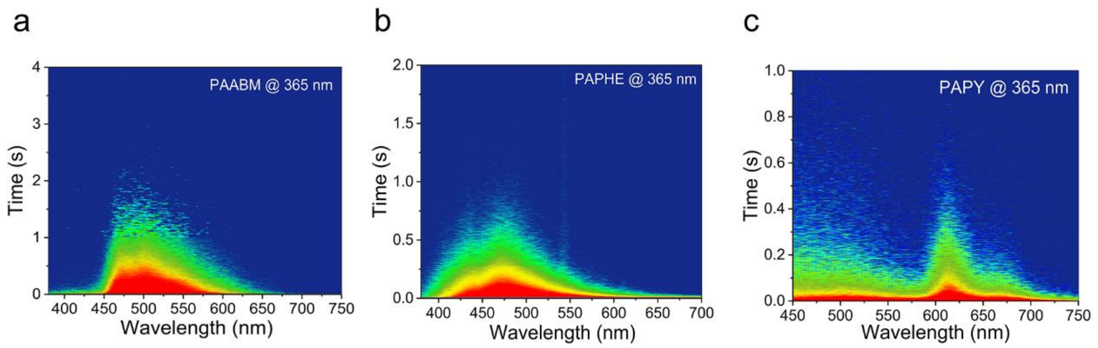

Supplementary Figure 26. a-c Time-resolved phosphorescence emission spectra of different single component polymer films PAABM (**a**), PAPHE (**b**) and PAPY (**c**) at room temperature under 365 nm excitation.

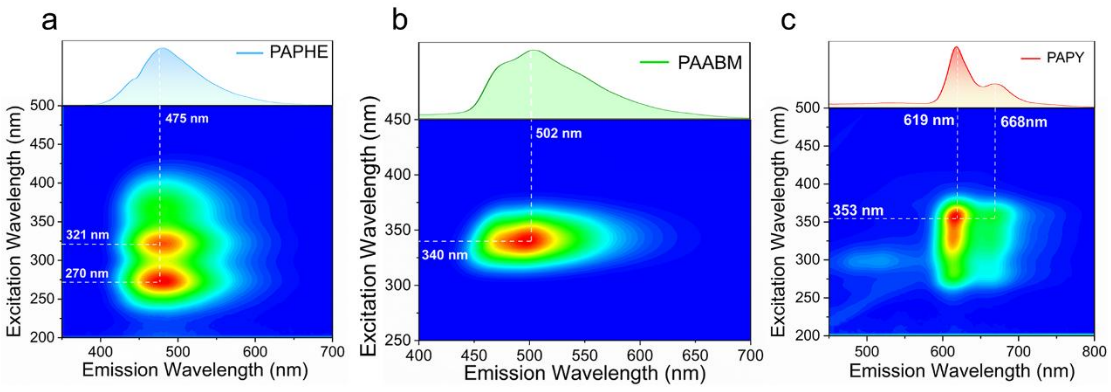

Supplementary Figure 27. a-c Excitation-emission phosphorescence mapping of polymer films PAPHE (excitation wavelength from 200 to 500 nm, emission wavelength from 350 to 700 nm) (**a**), PAABM (excitation wavelength from 250 to 500 nm, emission wavelength from 400 to 700 nm) (**b**) and PAPY (excitation wavelength from 200 to 500 nm, emission wavelength from 450 to 800 nm) (**c**).

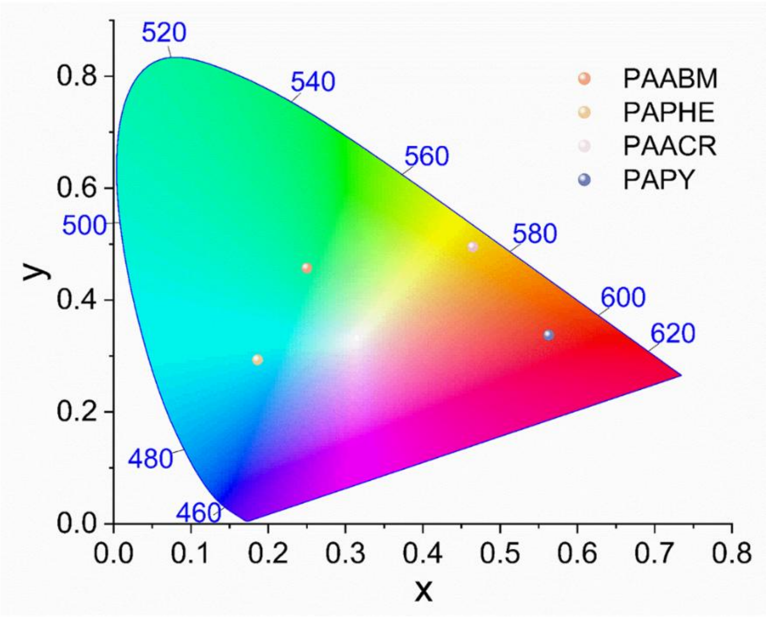

Supplementary Figure 28. CIE coordinates of the phosphorescence spectra of different one-component polymers PAABM, PAPHE, PAACR, PAPY excited at 365 nm were plotted.

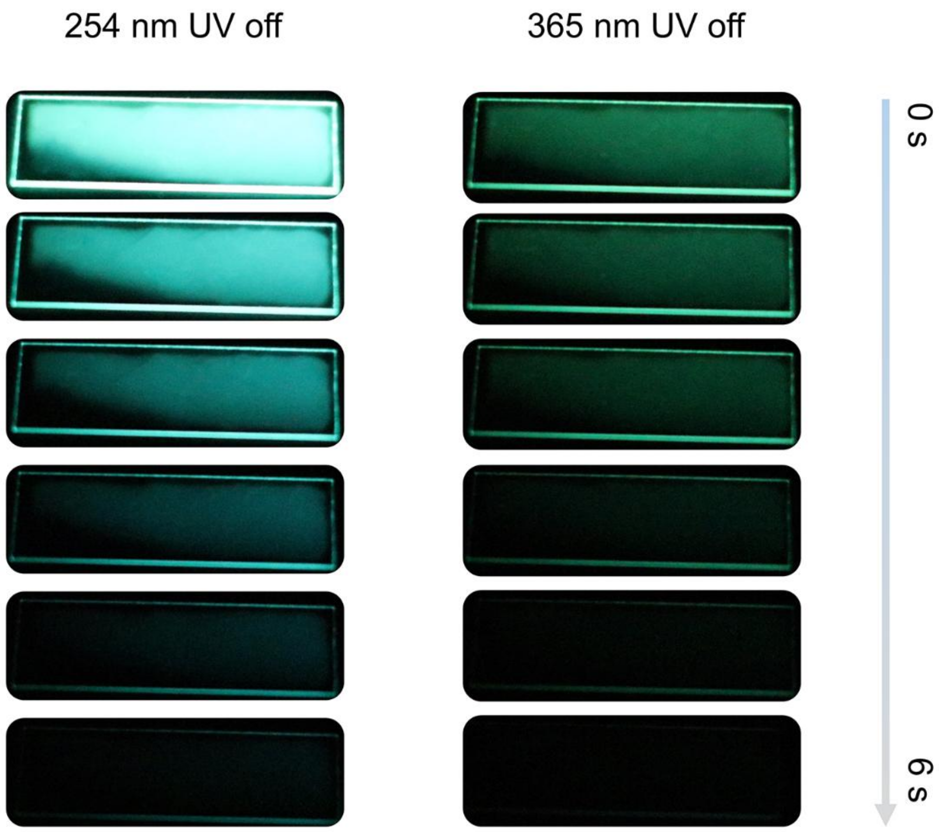

Supplementary Figure 29. Afterglow images of PAABM films excited by 254 nm and 365 nm under ambient conditions.

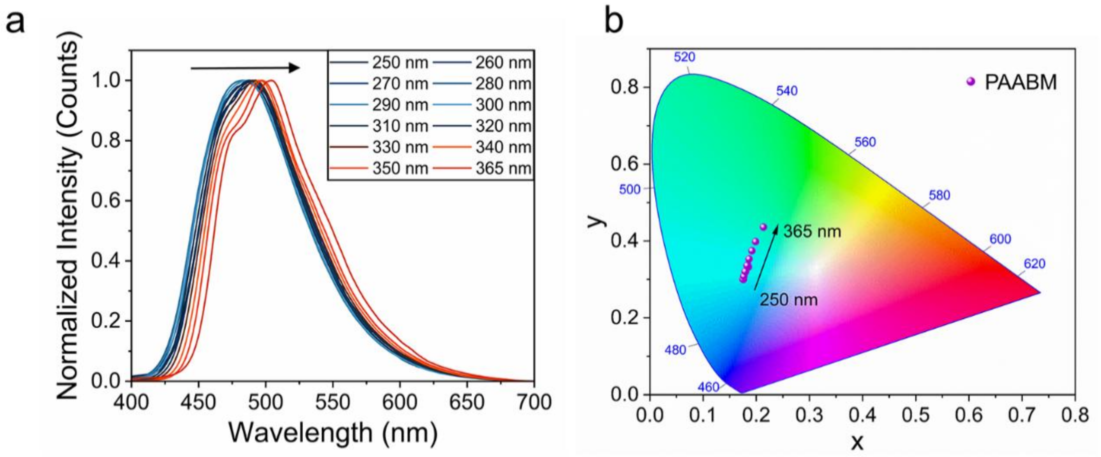

Supplementary Figure 30. **a** Phosphorescence spectra of PAABM films excited by different wavelengths under environmental conditions. **b** CIE coordinate diagrams corresponding to the phosphorescence spectra of PAABM films under different excitation wavelengths under environmental conditions. (λ_{ex} from 250 nm to 365 nm).

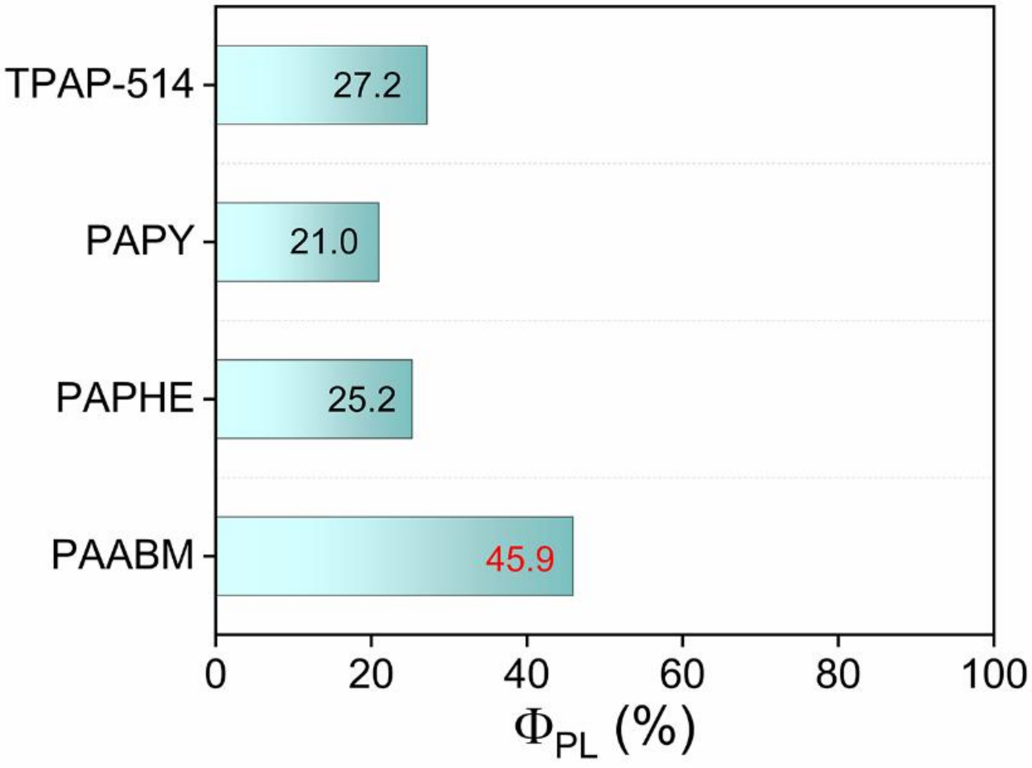

Supplementary Figure 31. Photoluminescence quantum yield of single-component polymer and white light emitter at room temperature.

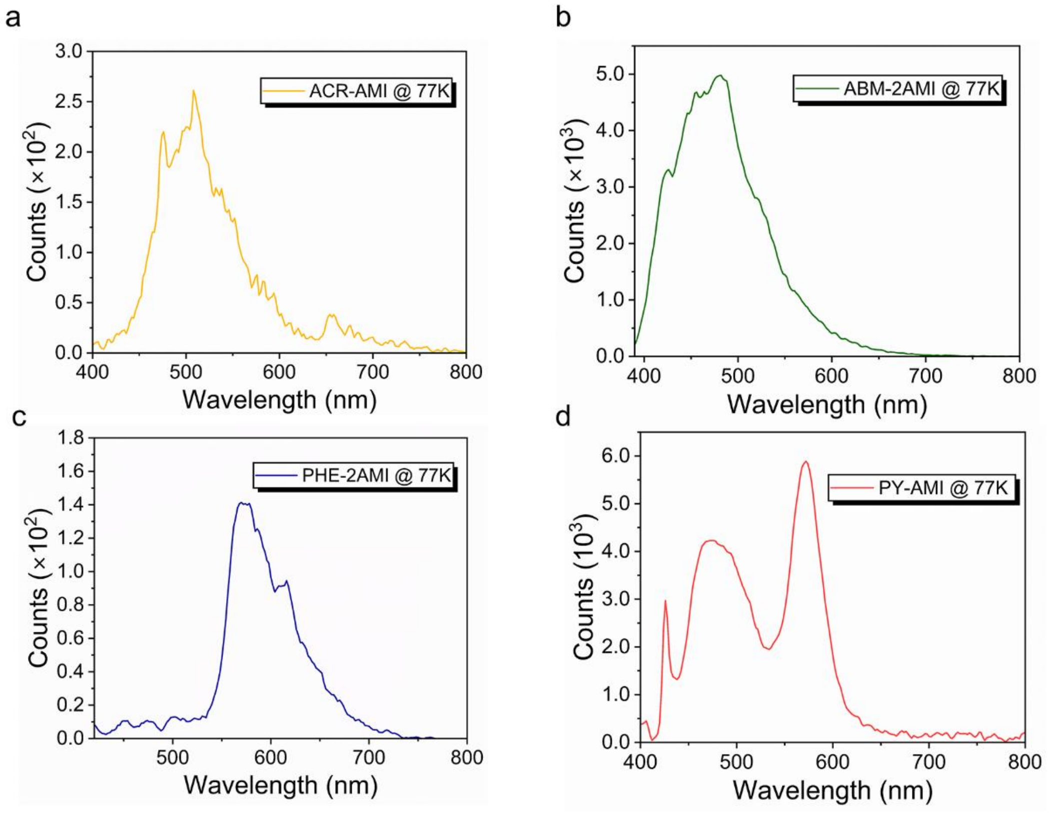

Supplementary Figure 32. a-d Powder low temperature (77 K) phosphorescence spectra of small molecules ACR-AMI (**a**), ABM-2AMI (**b**), PHE-2AMI (**c**) and PY-AMI (**d**) in phosphors.

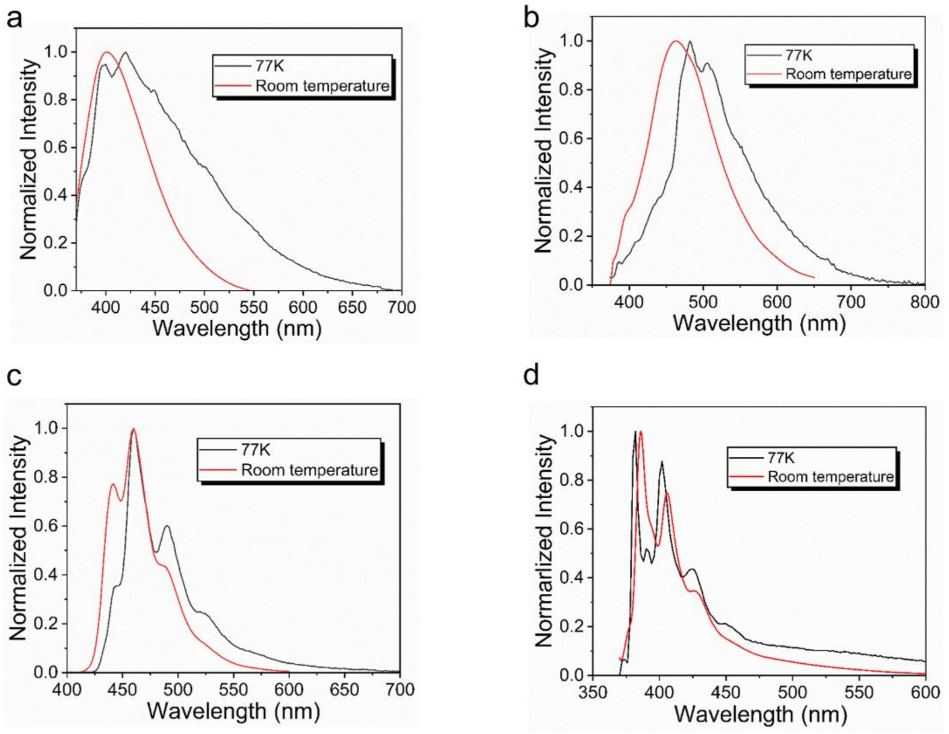

Supplementary Figure 33. a-d Fluorescence spectra of single-component polymers PAABM (**a**), PAPHE (**b**), PAACR (**c**) and PAPY (**d**) at 77 K and room temperature were excited at 365 nm.

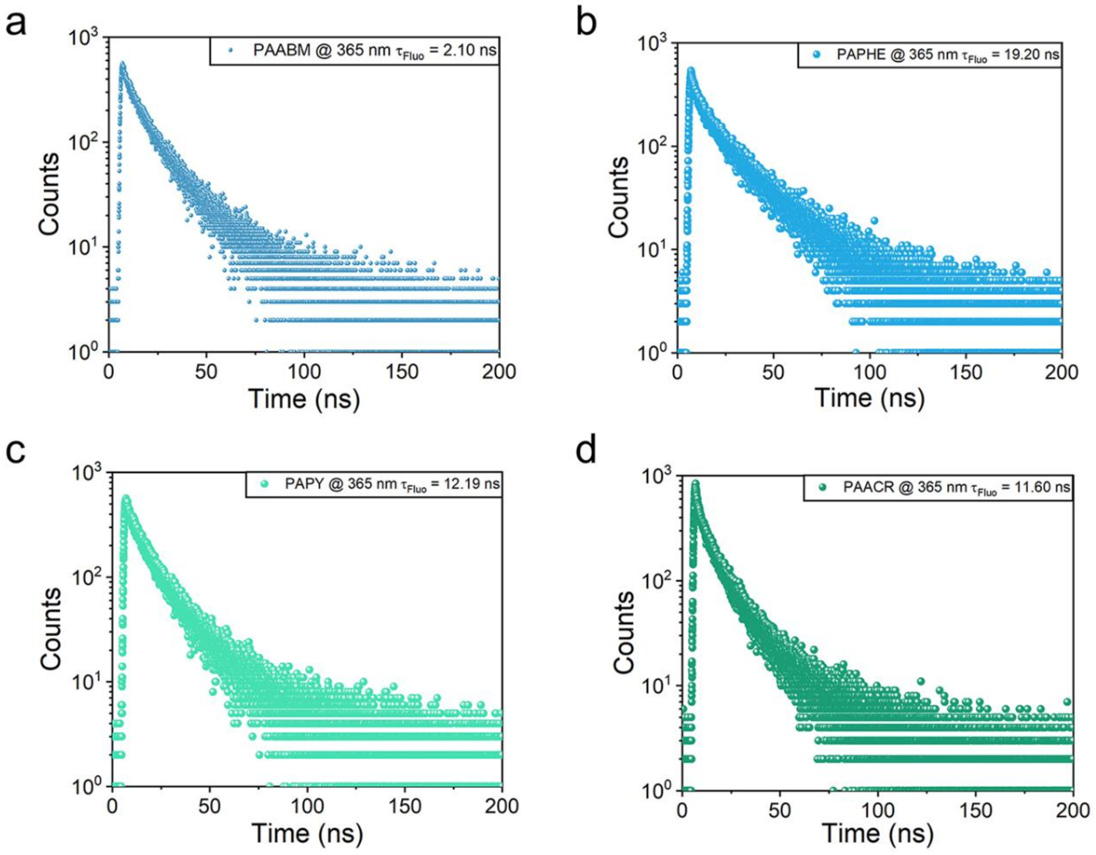

Supplementary Figure 34. a-d Fluorescence decay curve of the single component polymers PAABM (**a**), PAPHE (**b**), PAPY (**c**) and PAACR (**d**) at room temperature under 365 nm excitation.

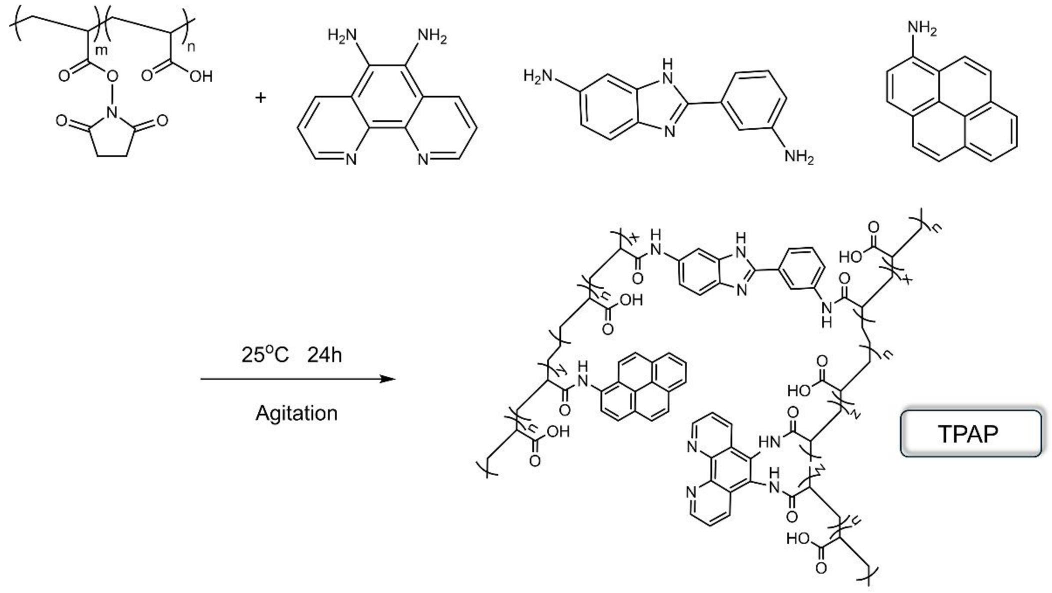

Supplementary Figure 35. Synthesis route of three-component polymer.

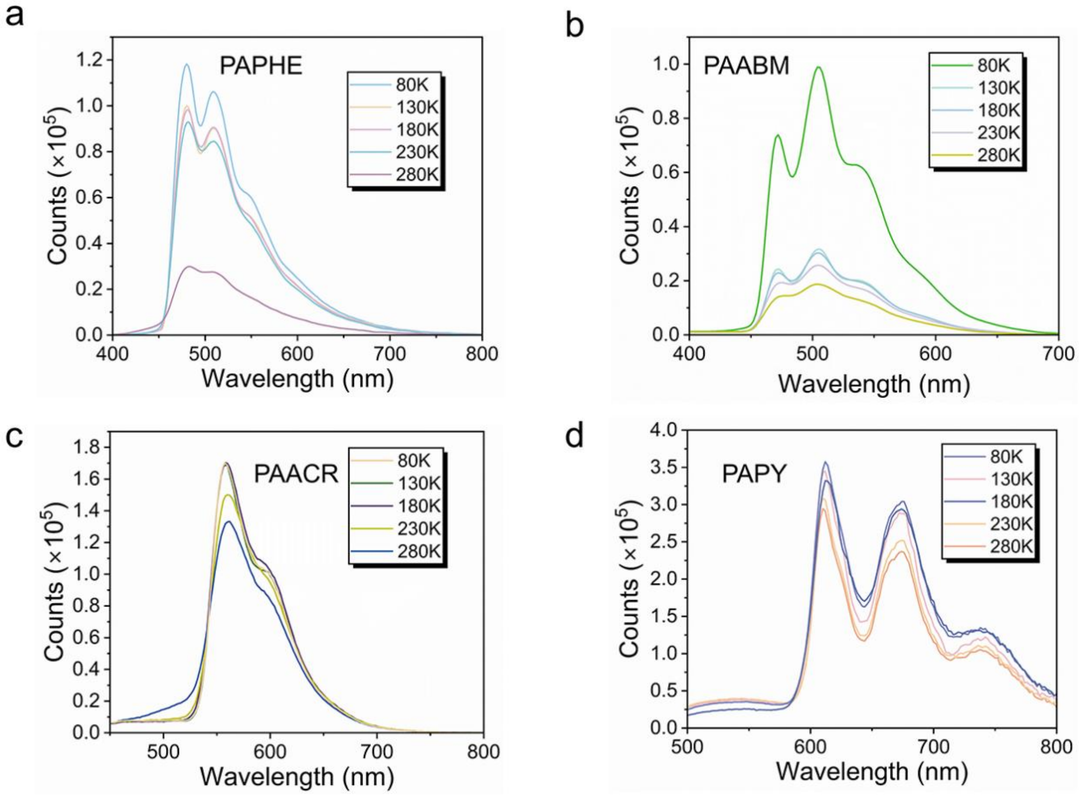

Supplementary Figure 36. a-d Phosphorescence spectra of PAPHE (**a**), PAABM (**b**), PAACR (**c**) and PAPY (**d**) films excited at 365 nm in the temperature range of 80-280 K.

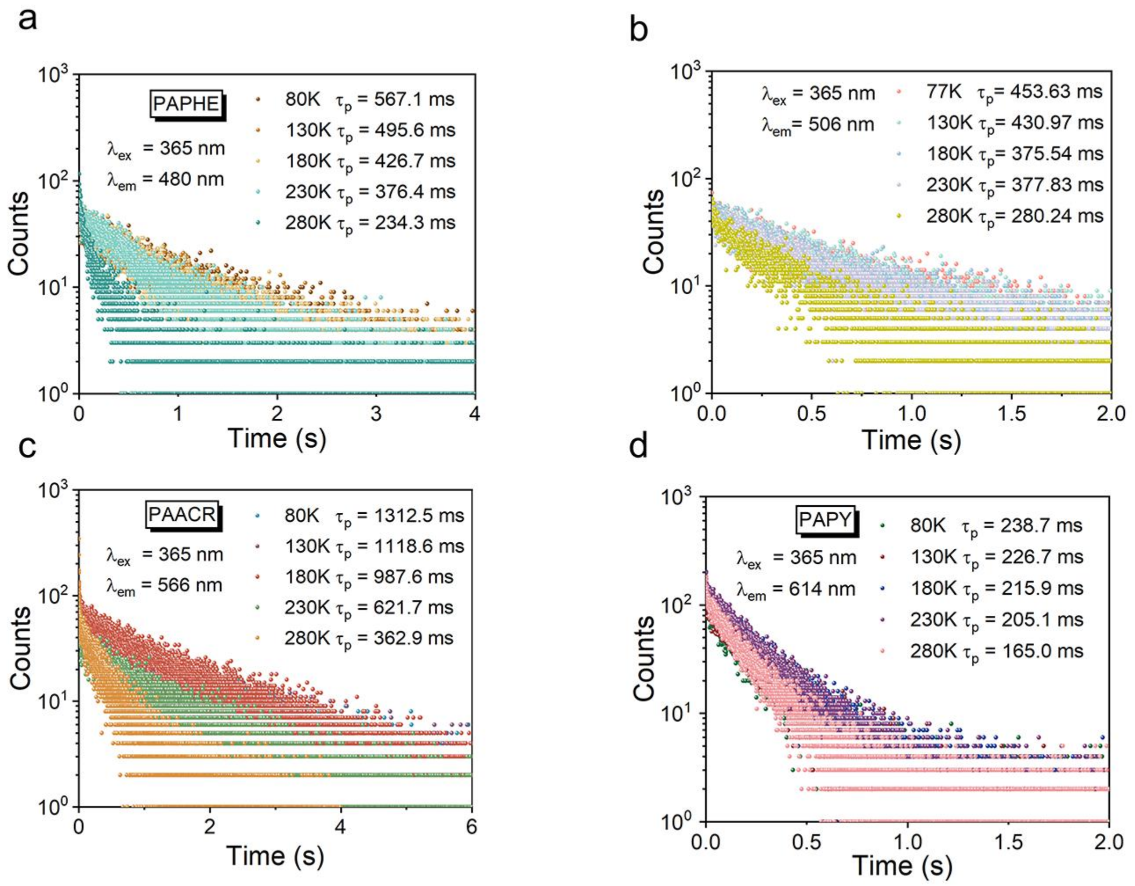

Supplementary Figure 37. a-d Phosphorescence decay curves of PAPHE (**a**), PAABM (**b**), PAACR (**c**) and PAPY (**d**) films excited at 365 nm in the temperature range of 80-280 K.

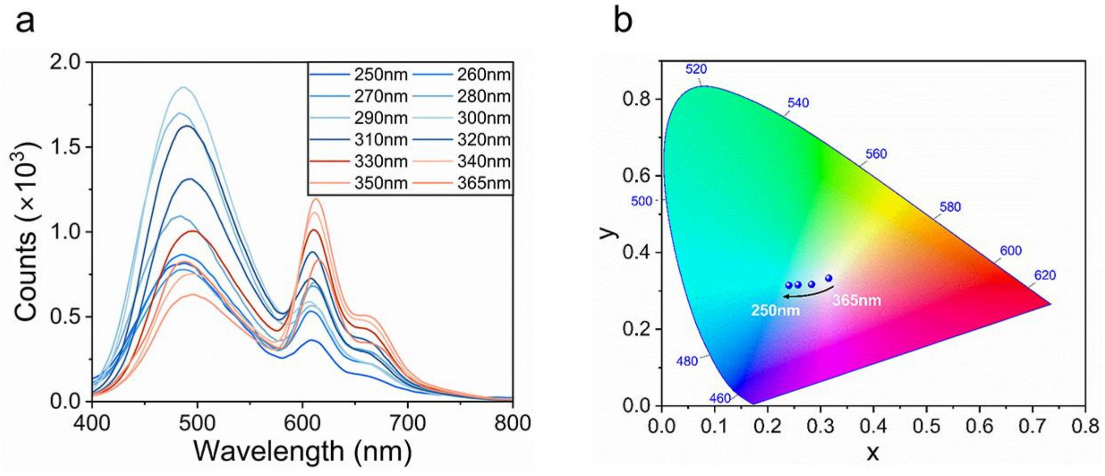

Supplementary Figure 38. a Phosphorescence spectra of TPAP-514 films excited by different wavelengths under environmental conditions. **b** CIE coordinate diagrams corresponding to the phosphorescence spectra of TPAP-514 films under different excitation wavelengths under environmental conditions (λ_{ex} from 250 nm to 365 nm).

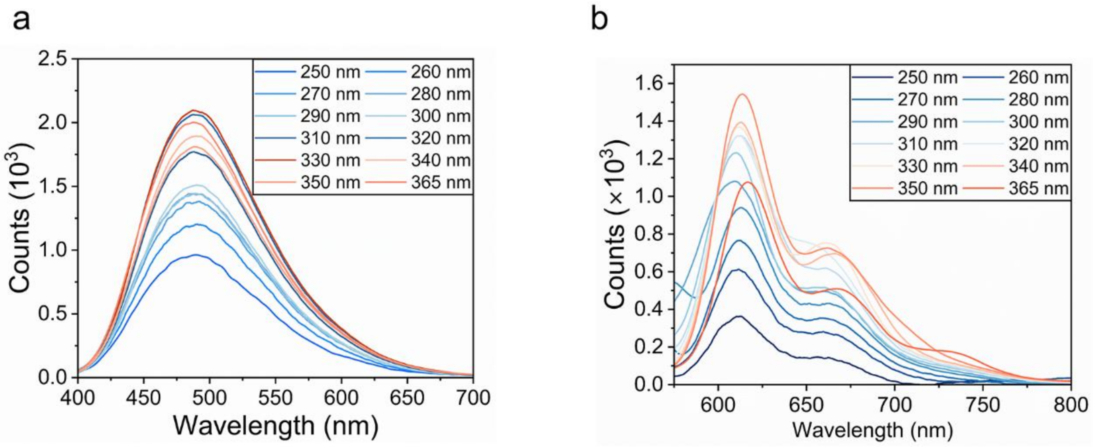

Supplementary Figure 39. a,b Phosphorescence spectra of polymer films PAPHE (**a**), PAPY (**b**) excited by different wavelengths under environmental conditions.

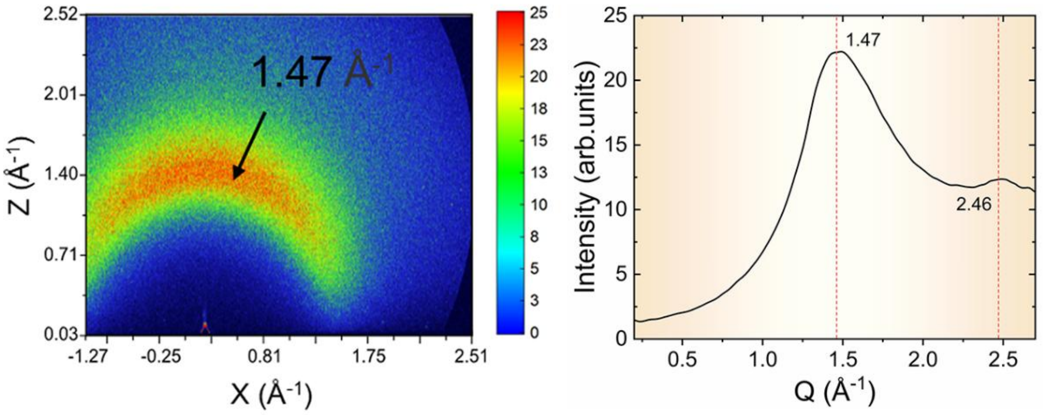

Supplementary Figure 40. GIWAXS pattern of TPAP-514 film.

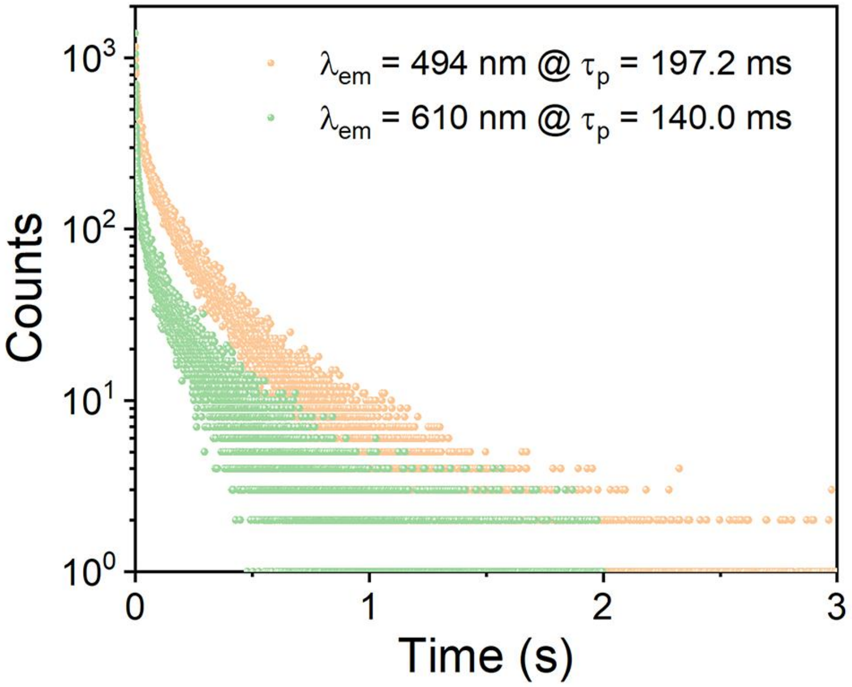

Supplementary Figure 41. Phosphorescence decay diagram of TPAP-514 film at 494 nm under environmental conditions.

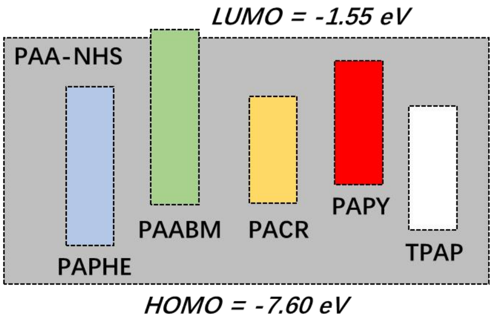

structure	HOMO (eV)	LUMO (eV)
PAA-NHS	-7.60	-1.55
PAPHE	-6.24	-1.83
PAABM	-5.56	-1.45
PAACR	-5.45	-1.93
PAPY	-5.24	-1.64
TPAP	-5.85	-2.03

Supplementary Figure 42. The highest occupied molecular orbital and the lowest occupied molecular orbital of single component polymer.

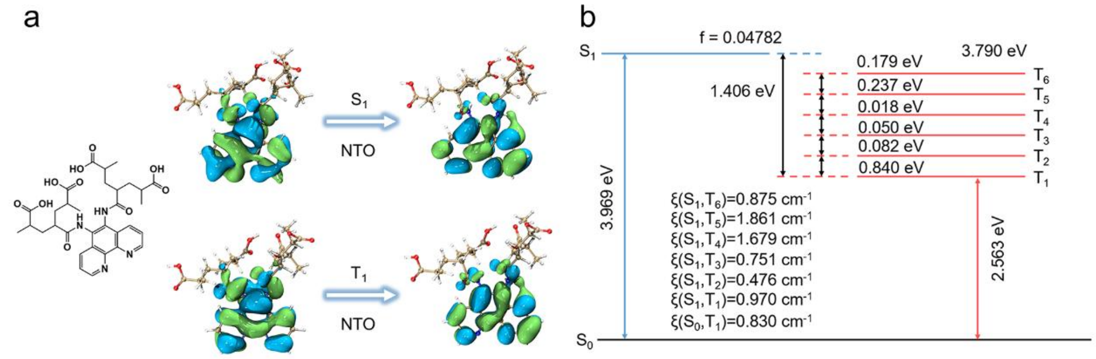

Supplementary Figure 43. Theoretical calculation of the polymer PAPHE by time-dependent DFT**. a** Natural transition orbitals of singlet and triplet excited states of PAPHE. **b** Energy levels and spin-orbit coupling constants of PAPHE.

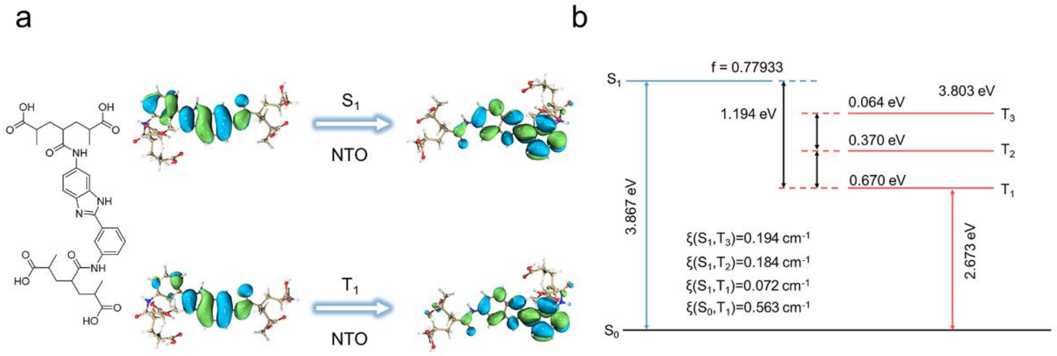

Supplementary Figure 44. Theoretical calculation of the polymer PAABM by time-dependent DFT**. a** Natural transition orbitals of singlet and triplet excited states of PAABM. **b** Energy levels and spin-orbit coupling constants of PAABM.

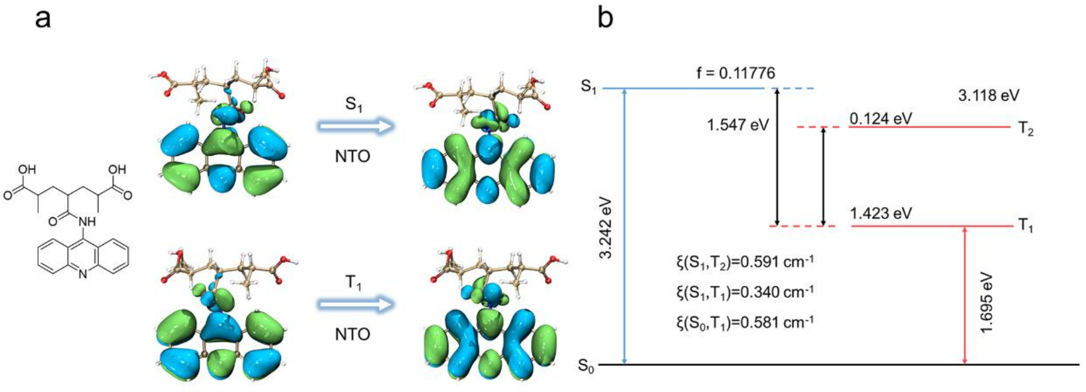

Supplementary Figure 45. Theoretical calculation of the polymer PAACR by time-dependent DFT**. a** Natural transition orbitals of singlet and triplet excited states of PAACR. **b** Energy levels and spin-orbit coupling constants of PAACR.

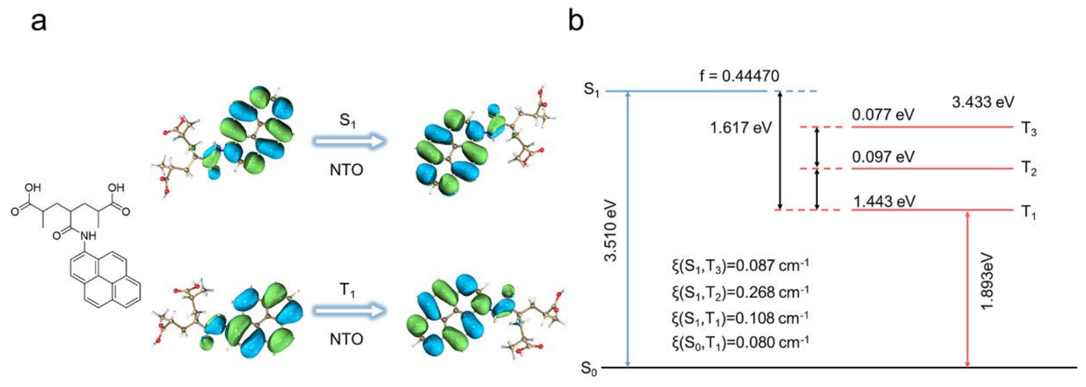

Supplementary Figure 46. Theoretical calculation of the polymer PAPY by time-dependent DFT**. a** Natural transition orbitals of singlet and triplet excited states of PAPY. **b** Energy levels and spin-orbit coupling constants of PAPY.

Supplementary Tables

Supplementary Table 1. GPC data of polymers using DMSO as the mobile phase.

System	Mn	Mw	Mp	Mz	Polydispersity	Mz/Mw	Mz+1/Mw
TPAP-514	40700	95160	81055	174506	2.34	1.83	2.79
PAPHE	44248	103408	88287	189813	2.34	1.84	2.80
PAABM	18747	41250	35036	76097	2.20	1.84	2.98
PAACR	20891	44696	39376	79067	2.15	1.77	2.75
PAPY	16331	38447	33735	71925	2.35	1.87	3.05

Supplementary Table 2. Photophysical data of single component polymers, where k_{nr}^{Phos} is calculated from Formula (1) $k_{nr}^{Phos} = (1\text{-}\ \Phi_{Phos})/\tau_{Phos}$

Sample	λ_{ex} (nm)	λ_{em} (nm)	τ_{Phos} (ms)	Φ_{Phos} (%)	k_{nr}^{Phos} (s^{-1})
PAPHE	270	475	334.7	14.7	2.54
PAABM	340	502	326.2	4.1	2.94
PAACR-15	364	566	396.9	10.0	2.27
PAPY	353	619	206.5	2.0	4.75

Supplementary Table 3. Photophysical data of three-component polymers with different feed ratios under environmental conditions, where k_{nr}^{Phos} and k_{r}^{Phos} are calculated from Formula (1) and (2) $k_{r}^{Phos} = \Phi_{Phos}/\tau_{Phos}$

Sample	τ_{Phos} (ms)	Φ_{PL} (%)	k_{r}^{Phos} (s^{-1})	k_{nr}^{Phos} (s^{-1})
TPAP-118	242.8	3.9	1.60×10^{-1}	3.96
TPAP-217	334.6	4.8	1.43×10^{-1}	2.76
TPAP-316	270.9	7.5	2.77×10^{-1}	3.42
TPAP-514	197.2	3.3	1.67×10^{-1}	4.90
TPAP-712	100.5	2.1	2.10×10^{-1}	9.75
TPAP-811	172.7	1.5	8.67×10^{-2}	5.70
