# Supplementary material for: Long lifetimes white afterglow in slightly crosslinked polymer systems
Source: Nat Commun. 2024 Apr 5;15:2947. doi: 10.1038/s41467-024-47378-2 (PMC10997626; doi:10.1038/s41467-024-47378-2)
Supplement: Supplementary file 3 — Description of additional supplementary files [file 41467_2024_47378_MOESM3_ESM.pdf]

DESCRIPTION OF ADDITIONAL SUPPLEMENTARY FILES

Supplementary Movie 1: Yellow phosphorescence of PAACR film under 365 nm UV excitation at room temperature. The power of excitation source is 30W.

Supplementary Movie 2: Long-lived room temperature phosphorescence video of PAACR film under visible light excitation. The power of excitation source is 10W.

Supplementary Movie 3: Blue phosphorescence of PAPHE film under 365 nm UV excitation at room temperature. The power of excitation source is 30W.

Supplementary Movie 4: Green phosphorescence of PAABM film under 365 nm UV excitation at room temperature. The power of excitation source is 30W.

Supplementary Movie 5: Red phosphorescence of PAPY film under 365 nm UV excitation at room temperature. The power of excitation source is 30W.

Supplementary Movie 6: The blue green and green phosphorescence of PAABM films excited by 254 nm and 365 nm ultraviolet light at room temperature. The excitation source power is 5W.

Supplementary Movie 7: White room temperature phosphorescence of three component polymer white emitter TPAP514 under 365 nm UV excitation. Irradiation about 6 s.
